# Easy Modular Integrative fuSion-ready Expression (Easy-MISE) Toolkit for Fast Engineering of Heterologous Productions in *Saccharomyces cerevisiae*

**DOI:** 10.1021/acssynbio.3c00015

**Published:** 2023-04-14

**Authors:** Letizia Maestroni, Pietro Butti, Riccardo Milanesi, Stefania Pagliari, Luca Campone, Immacolata Serra, Paola Branduardi

**Affiliations:** Department of Biotechnology and Biosciences, University of Milano-Bicocca, Piazza della Scienza 2, 20126 Milan, Italy

**Keywords:** synthetic biology toolkit, ready-for-fusion modular cloning, CRISPR-Cas9 marker-free genome editing, Saccharomyces cerevisiae, pathway engineering

## Abstract

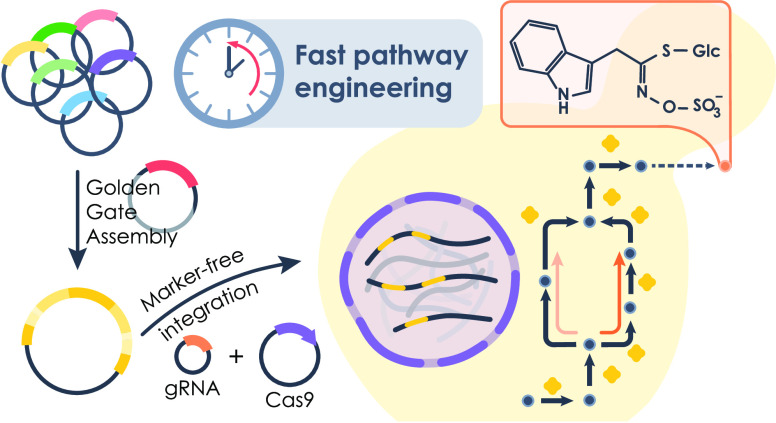

Nowadays, the yeast *Saccharomyces cerevisiae* is the platform of choice for demonstrating the proof of concept of the production of metabolites with a complex structure. However, introducing heterologous genes and rewiring the endogenous metabolism is still not standardized enough, affecting negatively the readiness-to-market of such metabolites. We developed the Easy Modular Integrative fuSion-ready Expression (Easy-MISE) toolkit, which is a novel combination of synthetic biology tools based on a single Golden Gate multiplasmid assembly meant to further ameliorate the rational predictability and flexibility of the process of yeast engineering. Thanks to an improved cloning screening strategy, double and independent transcription units are easily assembled and subsequently integrated into previously characterized loci. Moreover, the devices can be tagged for localization. This design allows for a higher degree of modularity and increases the flexibility of the engineering strategy. We show with a case study how the developed toolkit accelerates the construction and the analysis of the intermediate and the final engineered yeast strains, leaving space to better characterize the heterologous biosynthetic pathway in the final host and, overall, to improve the fermentation performances. Different *S. cerevisiae* strains were built harboring different versions of the biochemical pathway toward glucobrassicin (GLB) production, an indolyl-methyl glucosinolate. In the end, we could demonstrate that in the tested conditions the best-producing strain leads to a final concentration of GLB of 9.80 ± 0.267 mg/L, a titer 10-fold higher than the best result previously reported in the literature.

## Introduction

Synthetic biology is nowadays essential for the effective development of microbial cell factories able to produce heterologous molecules difficult to obtain through other processes. The yeast *S. cerevisiae* is an election chassis for these purposes, as demonstrated by several examples of heterologous production processes of complex molecules carried out in this final host, some of which have achieved the industrial production scale.^1,2^

One of the advantages of using *S. cerevisiae* as chassis is that its genetic manipulation is greatly facilitated by many synthetic biology tools.^3^ To reduce the readiness-to-market of metabolites deriving from long pathways and resulting in complex structures, there is a need to further expand and merge various synthetic biology approaches, to facilitate the applicability of the design–build–test–learn cycle and to further ameliorate the predictability of microorganisms’ engineering.

Two key principles of synthetic biology are modularity and reusability of built parts, which has to be designed to be used easily and flexibly. Therefore, the parts, DNA fragments with different purposes and origins (promoters, terminators, coding sequences, etc.), can then be assembled in complex devices, like expression cassettes, which, in turn, can then be used to engineer the final host. The possibility of assembling parts and building devices is offered by the variety of assembly methods, which allows joining many different DNA sequences in the desired order in a one-pot reaction with high efficiencies.^4^ Moreover, technological breakthroughs have made genome editing significantly easier, mainly thanks to the development of the CRISPR-Cas9 platform, which provides a powerful tool for sequence-specific genome editing, including gene knockout, gene knock-in, and site-specific sequence mutagenesis and corrections.^5^

It is important to notice that each system brings elements of novelty and ameliorations, and at the same time, its application indicates limitations and suggests further improvements.

Lee and colleagues^6^ recently developed a library of Golden Gate-assembled parts leading to plasmids, which then can be integrated into the *S. cerevisiae* genome. To do this, the authors made use of the CRISPR-Cas9 approach, exploiting the selectable markers included in the final plasmids to screen positive clones. The use of dominant and auxotrophic markers, brings with it some complications and limitations. Indeed, auxotrophic markers require the use of auxotrophic strains, limiting the genome editing possibilities, while dominant markers increase the process costs.

In a subsequent work by Jessop-Fabre and colleagues, the authors developed the so-called EasyClone-MarkerFree toolkit,^7^ which exploits USER cloning as a method to obtain new expression cassettes and the CRISPR-Cas9 genome editing method to engineer *S. cerevisiae* without the use of any selection marker. USER cloning is a costly strategy to be applied daily as it requires long uracil-containing primers, which are generally more expensive than regular primers. Moreover, since it is PCR based, it requires sequence verification of each final construct.

The approach proposed in this work combines the simplicity of design and construction of small integrative expression cassettes proposed in Jessop-Fabre et al.^7^ with the modularity and reusability of parts granted by Golden Gate Assembly, as in MoClo.^6^ The Easy Modular Integrative fuSion-ready Expression toolkit (Easy-MISE toolkit) was developed as an amelioration of previously mentioned synthetic biology toolkits and consists of a combination of synthetic biology approaches that embraces all of the principles described above, simplifying and accelerating the construction of chassis carrying different variants of heterologous pathways. It is characterized by a reduced design complexity combined with high modularity and flexibility thanks to a single Golden Gate Assembly multiplasmid reaction to obtain the final double transcription unit cassette. This device can then be integrated into the *S. cerevisiae* genome without using any marker, exploiting the CRISPR-Cas9 system and previously well-defined genome loci.^7^ The building of the ready-to-use library of parts and the final devices is accelerated thanks to the use of two new acceptor vectors, which allow color-based screening. Another feature proper of the toolkit is that it allows an easy in-frame tag of every ORF of interest (fusion-ready). This specific property has many applications and possible advantages. For example, it can be exploited to assess protein expression and localization in the cell exploiting an in-frame tag with a fluorescent protein.

To prove the applicability, practicality, and advantages of our novel combination of synthetic biology approaches, different *S. cerevisiae* strains were built to improve glucobrassicin production in yeast cell factories. Glucobrassicin (GLB), an indolyl-methyl glucosinolate, is the precursor of indole-3-carbinol, one of the most characterized bioactive derivatives of glucosinolates.^8−10^ Glucosinolates are secondary metabolites naturally produced by members of cruciferous vegetables as protective molecules against injuries and parasites, mainly thanks to their hydrolysis products. Interestingly, in humans, these products have been demonstrated to have cancer-preventive properties,^11^ raising interest in their exploitation as nutraceuticals or as additives in functional food. GLB heterologous production has been proven to be feasible in bakers’ yeast by two independent studies,^12,13^ comprising different pathways and enzymatic variants, opening further investigation and possible optimization. The Easy-MISE toolkit allowed a quick and easy expression of the two versions of the GLB pathway reported in the literature. First, the two possible biosynthetic routes were reconstructed by exploiting the “Modular and Integrative” features of the toolkit. Then, GLB production was compared, and the best version of the pathway was finally defined. Furthermore, thanks to the flexibility of the tool, the contribution of the two different homologs of cytochrome CYP79B2, the first enzyme of the pathway, was compared, one from *Brassica oleracea* var. *botrytis* and the other from *Arabidopsis thaliana*. Finally, the “fuSion-ready” module of the toolkit was exploited to tag each coding sequence with GFP, to verify their translation and localization. By combining the best results obtained, a strain that reached a GLB titer of 9.80 ± 0.267 mg/L, 10 times higher than what was previously reported, was built, showing the importance of testing different orthologues of the key pathway enzymes. These achievements confirm the potential of the synthetic toolkit in accelerating the amelioration of microbial cell factories and their productive performances.

## Results and Discussion

### Easy-MISE Toolkit

#### General Overview of the Easy-MISE Toolkit

**Figure 1:**
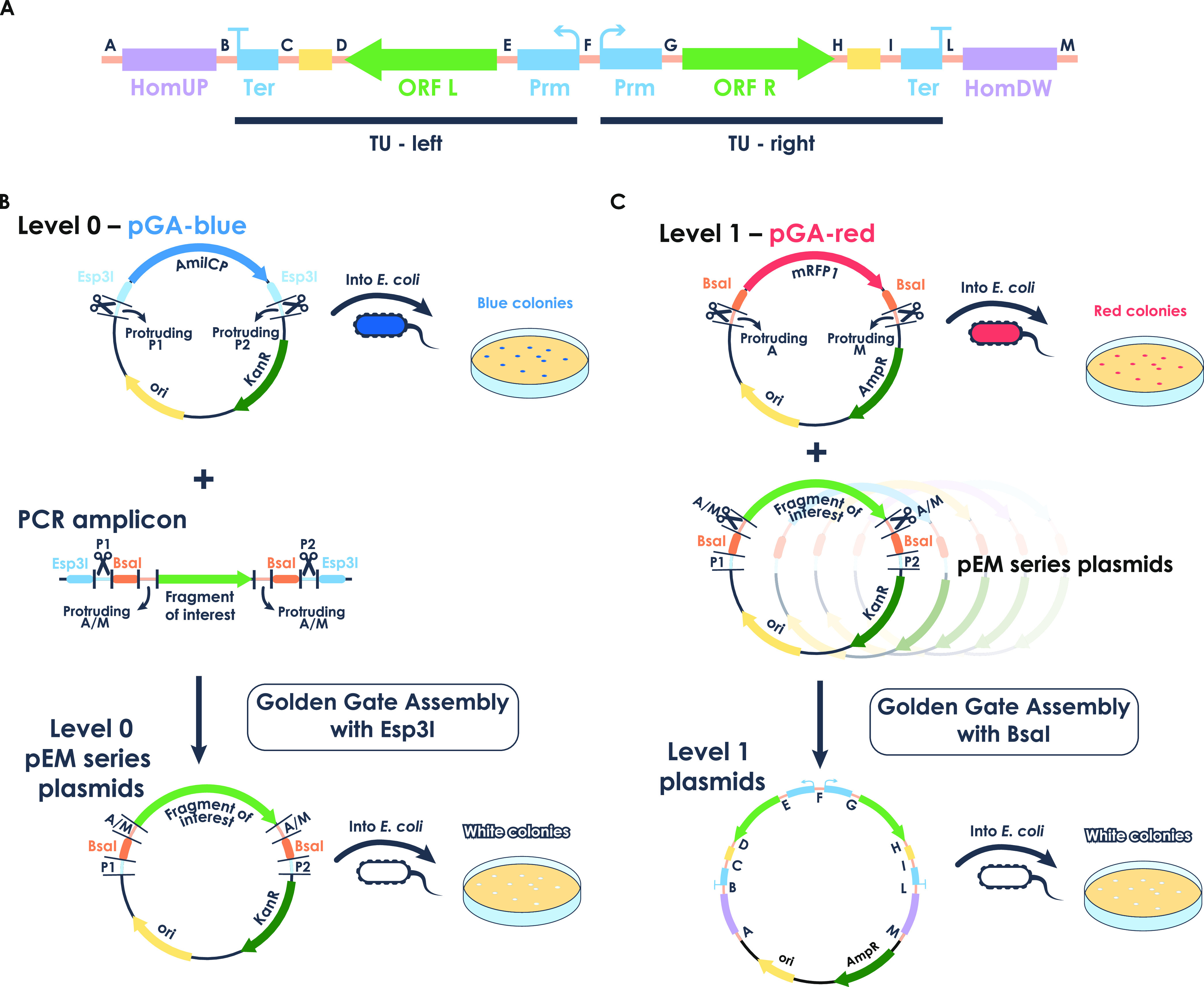
Easy-MISE toolkit: construction of level 0 and level 1 plasmids. (A) Easy-MISE toolkit final integration cassette consists of two divergent symmetrical transcription units. TUs are composed of a promoter (Prm), an ORF (L, left and/or R, right), an in-frame-fusion part (tag or adaptor, yellow rectangle), and a terminator (Ter); the TUs are flanked by homology regions for genomic integration. Parts cloning is obtained with the Golden Gate assembly reaction and allowed by 4 bp protruding sequences, represented in orange and indicated with the letters from A to M (Table 1). (B) Level 0 is obtained by cloning the fragment of interest into the pGA-blue acceptor plasmid. The plasmid carries an *E. coli* amilCP expression cassette, allowing for a blue/white screening system. The cloning is obtained by digesting pGA-blue and the fragment of interest with *Esp*3I sites, exploiting P1 and P2 protruding sequences for ligation. As a result, *Esp*3I cutting sites and amilCP are replaced with the fragment of interest flanked by *Bsa*I recognition sites (white colonies), obtaining the pEM series plasmid(s). (C) Likewise, level 1 is obtained with the pGA-red acceptor vector and a red/white screening system based on the expression of mRFP1 (red colonies). Level 1 plasmids are obtained with a Golden Gate reaction in which pEM series plasmids and the pGA-red acceptor are digested with *Bsa*I. A to M protruding sequences allow for the assembly of the desired double TU integration cassette into a pGA-red acceptor (white colonies).

In the present study, we designed and developed a Modular, Integrative, and fuSion-ready toolkit, the Easy-MISE toolkit, which is a novel combination of synthetic biology tools featuring a flexible design of a final double transcription unit cassette (Figure 1A).

The toolkit is based on a single Golden Gate multiplasmid assembly characterized by a chromoprotein-based screening. This strategy maximizes the probability of screening positive clones, reducing the number of tested colonies. This combination reduces design and construction time for cassette preparation while allowing a higher degree of modularity with respect to previously developed methods.^7^ Furthermore, an easily expandable library of Golden Gate Assembly parts allows high flexibility.

The second advantage is the design of the final integration cassette, shown in Figure 1A, to specifically integrate by homologous recombination in selected loci of the *S. cerevisiae* genome, previously identified for their stability and high transcription level.^7^ Each integration cassette contains an upstream and a downstream homology sequence for targeted genome insertion (HomUP and HomDW) and two transcription units (TUs), which are transcribed in opposite directions (therefore named left or right, TUL and TUR). Our design also allows building integration cassettes with only one TU since either TUL or TUR can be replaced with specific adaptors (Figure 1A).

The additional flexibility of the system (third advantage) stays in the “fusion-ready” module, which is designed to be inserted at the 3′ and in frame with the coding sequence, before the terminator. This part can be a tag useful for simply tracing the protein or for adding another functional moiety. In case a fusion element is not required, a classical assembly of the expression device can be completed, as the library provides adaptors comprising a stop codon that connect the ORF with the terminator.

With the Easy-MISE toolkit, it is possible to obtain strains with six ORFs integrated into the yeast genome every 4 weeks, and, depending on the need, it can be easily exploited for studying enzyme localization, alternative pathways, and identification of bottlenecks. In light of the described property, we strongly believe that the Easy-MISE toolkit represents a significant improvement of the available solutions for the metabolic engineering of *S. cerevisiae*.

#### Design and Build of the Easy-MISE Toolkit

The Easy-MISE pEM plasmid series constitutes the “level 0” of the toolkit in which parts are cloned (Figure 1B). The existing parts consist of integration homology, promoters, terminators, adaptors, and fluorescent proteins. All of these parts create the library of level 0 plasmids of the Easy-MISE toolkit called pEM (partEasyMise) plasmids, which can be combined to build the expression cassettes. All pEM plasmids are listed in Table S3.

The system used in this work relies on a set of six different promoters with different expression levels, based on the work of Peng and co-workers,^14^ namely, *pENO2*, *pTDH3*, *pTPI1*, *pPGK1*, *pPDA1*, and *pCYC1*. Regarding the terminators, the toolkit presents the widely used *tADH1* for the TUL and *tCYC1* for the TUR, while the homology regions were built considering the integration sites described in the work of Jessop-Fabre and colleagues.^7^

As described above, the toolkit allows for the in-frame tag at the C-terminal of every ORF of interest. This is achieved by the presence of a DNA spacer between each ORF and terminator that can be substituted with the desired sequence during the construction of the integration cassette (Figure 1A). Indeed, the ready-to-use library already comprehends GFP and mCherry coding sequences to be used as tags for the study of protein localization. A GFP version carrying an amino acidic linker of 10 residues is also present in case the direct in-frame fusion is not optimal for GFP folding. All of the ORFs of the toolkit must be consequently cloned in pEM plasmids without the stop codon to allow the in-frame fusion with protein domains of interest. Moreover, this feature is particularly useful for future implementations, considering studies suggesting that synthetic protein scaffolding and enzyme colocalization could help in the fine-tuning of pathways’ flux distribution.^15^ The integration cassettes are easy to be designed and built, assembled and reassembled in different versions, thanks to the newly designed acceptor vectors for Golden Gate assembly (Figure 1B,C).

The acceptor vector to build level 0 plasmids is named pGA-blue and is used to build the ready-to-use library of parts. It carries the blue amilCP chromoprotein that, once expressed in *Escherichia coli*, generates intensely blue colonies (Figure 1B).^16^ The cloning strategy is based on the loss of the amilCP chromoprotein and its substitution with the part of interest: blue clones are considered negative, while the positive clones will appear white (Figure 1B). On the one hand, thanks to the presence of *Esp*3I Type IIS restriction enzyme recognition sites at both ends of the amilCP coding sequence, *Esp*3I cleaves amilCP and generates the two protruding ends P1 and P2 (Table 1). On the other hand, the parts must be amplified by PCR using ad hoc-designed primers to obtain a final amplicon with specific ends. Figure 1B shows the structure of the final amplicon.

**Table 1:** Protruding Sequences Used to Design and Build the Different Parts of the Easy-MISE Toolkit and Selected from the Work of Potapov and Colleagues^17^^a^

P1	P2	A	B	C	D	E	F	G	H	I	L	M
TGGT	GGTC	TGCC	ACTA	CAGA	AACT	GAGC	AGGA	ATTC	ACCG	ATAG	TTAC	GCAA

a Using sets of well-characterized junction pairs avoids the creation of erroneous assemblies.

In order to build level 1 plasmids, we developed two different acceptor vectors accommodating the final integration cassette (Figure 1C), called pGA-red-maxi and pGA-red-mini, with different backbones (Figure S2). Both carry the red-fluorescence protein (mRFP1) chromophore, following the same selection strategy presented for the pGA-blue plasmid except for the fact that now negative clones will appear red (Figure 1C). The mRFP1 expression cassette is flanked by a *Bsa*I Type IIS restriction enzyme recognition site, and its cleavage generates A and M protruding ends (Table 1). Level 1 plasmids are built by cloning into pGA-red all of the designed parts, mixing in a single Golden Gate assembly reaction all of the required pEM plasmids and the desired acceptor vector (Figure 1C). To neatly assemble integration cassettes with fragments in the desired order, the four-base overhangs have been chosen from the work of Potapov et al^17^ (Table 1, A–M) (Figure 1C) as those were tested as high fidelity sets.

To obtain the final integration cassette to be used for yeast transformation, level 1 plasmids must be linearized with the *Nhe*I restriction enzyme. The purification feasibility of the integration cassette from the plasmid backbone depends on the difference between their lengths. The two pGA-red plasmids (maxi and mini) have been designed as two alternative options, differing in the length of the backbone (Figure S2): in this perspective, one or the other pGA-red acceptor vector can be chosen depending on the specific length of the final desired integration cassette. In addition, pGA-red-maxi bears an autonomously replicating sequence and a selection marker, making possible preliminary fast screening without genomic integration.

#### Workflow of the Easy-MISE Toolkit

**Figure 2:**
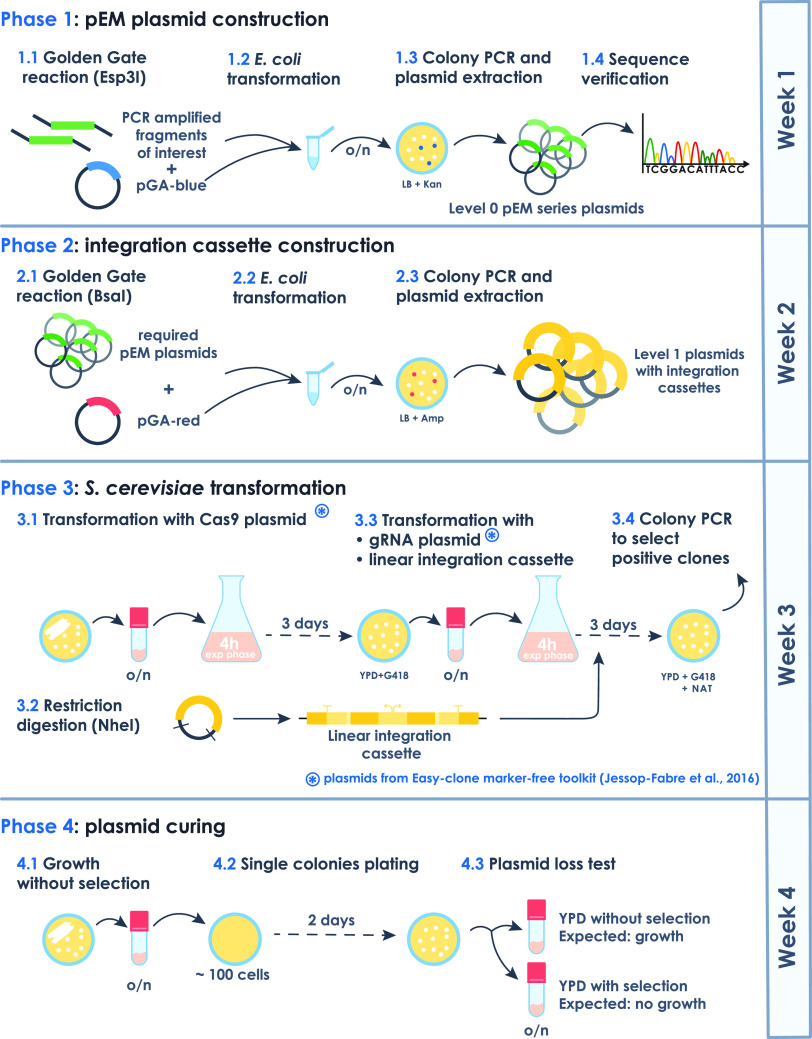
General workflow of the engineering of *S. cerevisiae* cells with the Easy-MISE toolkit.

Overall, adapting already existing cloning and integration strategies, we developed a linear and simple workflow for strain construction characterized by a reduced operational time and a higher level of reusability of intermediate strains and parts (Figure 2).

A schematic representation of the workflow is reported in Figure 2. Once the level 0 and level 1 plasmids are ready (phases 1 and 2), the integration cassettes harbored into the pGA-red acceptor vector are cut with the *Nhe*I restriction enzyme. The linear fragment is consequently used to transform the *S. cerevisiae* strain according to the “EasyClone-MarkerFree” toolkit and the manual^7^ (phase 3). Thanks to this tool, it is possible to exploit the CRISPR-Cas9 system without the use of selection markers and together with the use of predefined and well-characterized chromosomal targets to integrate the expression cassette of interest. The “EasyClone-MarkerFree” toolkit comprises both gRNA helper vectors for single chromosomal integration and gRNA helper vectors driving triple site targeting.^7^

This allows for the construction of a yeast strain containing from one to six expression cassettes in just one transformation (Figure 2).

The correct integration of double transcription units into the *S. cerevisiae* genome is verified by colony PCR. The percentage of positive integrations using single-gRNA plasmids spanned from 70 to 100% for all of the integration sites, except for the XI-2 locus, which showed an efficiency of 22% (Table S6). The different yeast genetic backgrounds between this work and the work of Jessop-Fabre and colleagues^7^ might be the cause for the difference in the transformation efficiencies in the XI-2 locus. The reduced integration efficiency for this single locus can also justify the very low multiple integration rate when it is targeted together with X-3 and XII-2 loci using a three-gRNA plasmid (Table S6), an event that already normally occurs at frequencies lower than those of single integrations. Screening a large number of colonies or a future redesign of gRNA targets can help in overcoming this limitation.

As the last step, the new strains must be plasmid curing to remove the gRNA helper vector and prepare the strain for the next round of transformation (phase 4). During the whole engineering procedure, the Cas9 expression vector is maintained with selective media and can be removed once the final strain is obtained.

### Glucobrassicin Biosynthetic Pathway in *S. cerevisiae*: A Proof of Concept

#### General Overview of the GLB Biosynthetic Pathway

**Figure 3:**
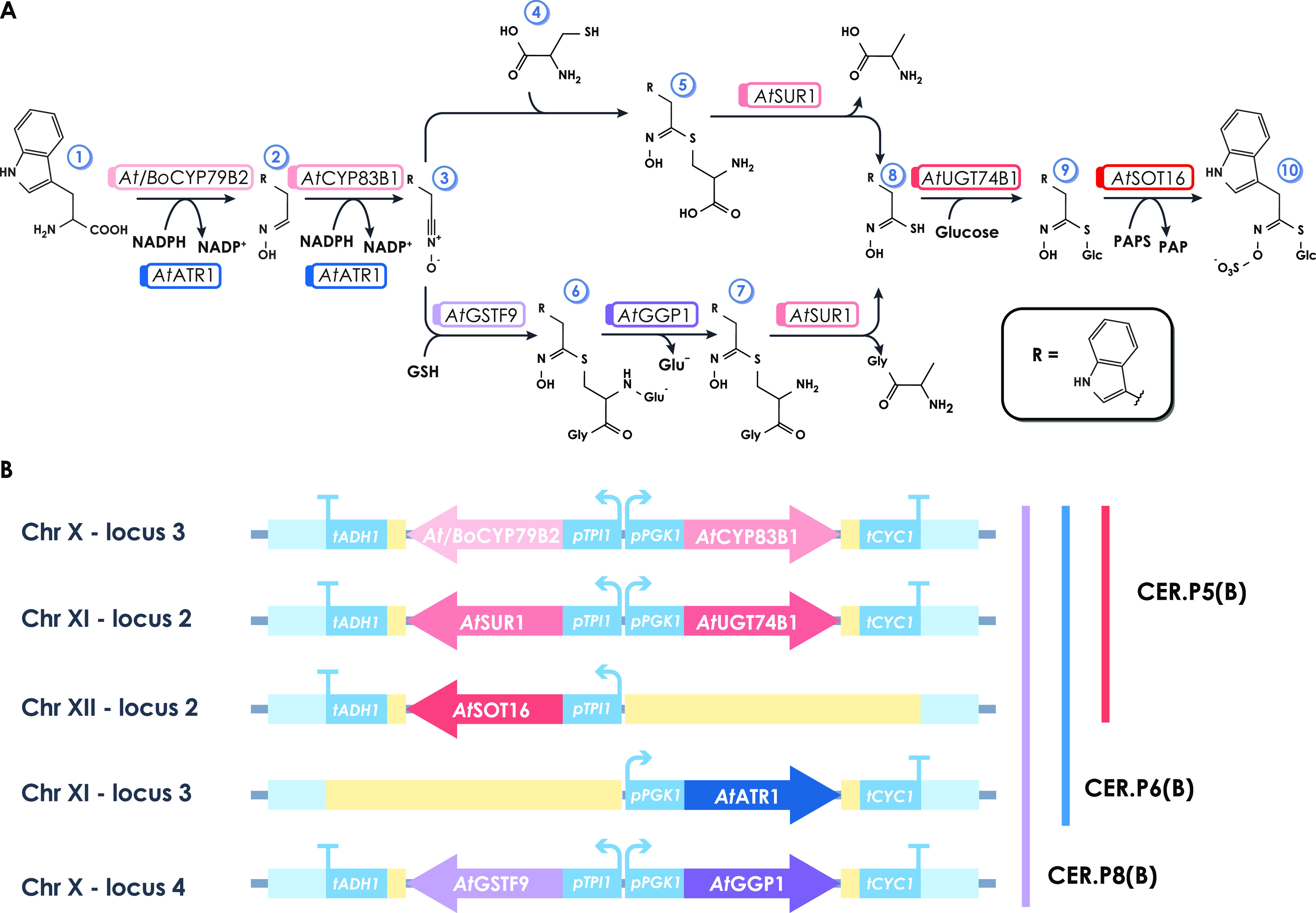
Glucobrassicin-producing pathways and -producing strain genotype. (A) The two alternative GLB biosynthetic pathways are described. The top branch relies on a spontaneous condensation of cysteine with indolyl acetonitrile oxide. The bottom branch is glutathione-dependent and enzymatically catalyzed. Genes and corresponding enzymes are indicated for each step. Numbers identify the following compounds: (1) tryptophan, (2) indolyl acetaldoxime, (3) indolyl acetonitrile oxide, (4) cysteine, (5) Cys conjugate (6) S-[(Z)-indolylacetohydroximoyl]-l-glutathione (GSH conjugate), (7) Cys–Gly conjugate, (8) indolyl acetothiohydroximic acid, (9) desulfoglucobrassicin, (10) glucobrassicin. (B) Yeast cells are modified to express different variants of the GLB biosynthetic pathway. The strain CER.P8 carries all of the eight coding sequences for the glutathione-dependent pathway from *A. thaliana*; CER.P8.B is built in an analogous manner, but it contains the coding sequence for the CYP79B2 cytochrome from *B. oleracea* var. *botrytis*. The strains CER.P6.B and CER.P5B express the CYP79B2 cytochrome from *B. oleracea* var. *botrytis* and the other enzymes of the cysteine-dependent pathway from *A. thaliana*, including or not (respectively) the ATR1 reductase. The colors of the coding sequence in panel (B) match the colored boxes of the enzymatic activities in panel (A), for easier interpretation of the genotype–phenotype correlation.

As previously introduced, two different variants of the biosynthetic pathway for the heterologous production of GLB are reported in the literature and presented here in Figure 3A. The cysteine-dependent pathway^12^ is composed of five enzymatic steps, including a spontaneous reaction between the indolyl acetonitrile oxide and the cysteine, the sulfur-donating molecule. Authors reported the possibility of obtaining GLB in an *S. cerevisiae* strain overexpressing five heterologous plant genes: two P450 cytochromes (*CYP79B2* and *CYP83B1*), a C-S-lyase (*SUR1*), a glucosyl-transferase (*UGT74B1*), and a sulfotransferase (*SOT16*). A later work achieved GLB production in yeast by adding two enzymatic steps to the previous ones at the node of the sulfur-donating step, a glutathione s-transferase (*GSTF9*) and a γ-glutamyl peptidase (*GGP1*), consuming the glutathione as a sulfur-donating molecule.^13^ Moreover, only these authors included the expression of ATR1 reductase due to its role in cytochrome P450-mediated metabolism. Overall, this second version of the pathway comprised the expression of eight heterologous genes, while for the first one, only five are needed.

The first study exploited coding sequences from *B. oleracea* var. *botrytis*, while the second used *A. thaliana*. Moreover, the two studies are based on different expression systems: multicopy episomal plasmids were used in the first, while genomic integration was used in the second, with different promoters (including strong inducible ones).

As the two works were not directly comparable and only for the second pathway a quantitative analysis was reported, the contribution of the sulfur-donating step and the ATR1 reductase in maximizing GLB production performances remained to be clarified.

In addition, in plants, glucosinolate metabolism starts with the oxidation of precursor amino acids by the CYP79 family cytochrome P450 monooxygenases. CYP79 monooxygenases differ in terms of substrate specificity and can direct the synthesis toward a specific glucosinolate:^18^ the different GLSs obtained at the end of the biosynthetic pathway strictly depend on the first cytochrome specificity and selectivity.^19,20^ This evidence, in combination with the work from Bartolucci et al.,^12^ led us to take advantage of plant enzymatic biodiversity at the level of the initial cytochrome CYP79B2.

To investigate these issues, we leveraged the flexibility of the Easy-MISE toolkit to rapidly design, build, and test yeast strains producing GLB through the two alternative pathways, testing two different orthologues of the pathway entry enzyme and assessing the role of cytochrome reductase.

#### “Modular and Integrative”: Designing and Building Glucobrassicin-Producing Strains

To validate the efficacy of the novel biomolecular tool, both versions of the GLB biosynthetic pathway were integrated into the *S. cerevisiae* genome, obtaining a panel of *S. cerevisiae* strains and allowing a better investigation of GLB production in yeast.

We built pEM plasmids carrying all of the plant ORFs coding for the 5- or 7-step pathway and for the ATR1 reductase. These plasmids, together with the needed pEM plasmids from the ready-to-use library (homology regions, promoters, terminators, and adaptors), were secondarily used in different Golden Gate reactions to build the double transcription units and obtain the level 1 plasmids (G5-8 and G11, Table S4).

The construction of the final strains tested for GLB production is described more in detail in the Supporting Information: Methods. In the end, CER.P8, CER.P8.B, CER.P5.B, and CER.P6.B strains were built. A schematic representation of the final genetic modification of the different strains is reported in Figure 3B, while in Table S1 all of the specific genotypes are reported. CER.P8 carries all eight coding sequences for the glutathione-dependent pathway from *A. thaliana*, while in CER.P8.B, the coding sequence for the CYP79B2 cytochrome is from *B. oleracea* var. *botrytis*. Accordingly, also CER.P5.B and CER.P6.B strains carry the *Bo*CYP79B2 cytochrome, while the other coding sequences for the cysteine-dependent pathway are from *A. thaliana*; the difference between these latter two strains is that CER.P6.B carries also the coding sequence for ATR1 reductase.

Thanks to the construction of the strains here presented, we were able, on the one hand, to elucidate the contribution of the sulfur-donating step and the ATR1 reductase and, on the other hand, to investigate the impact on the production of different cytochrome CYP79B2 homologs.

#### “Modular and Integrative”: Test and Learn from Glucobrassicin-Producing Strains

**Figure 4:**
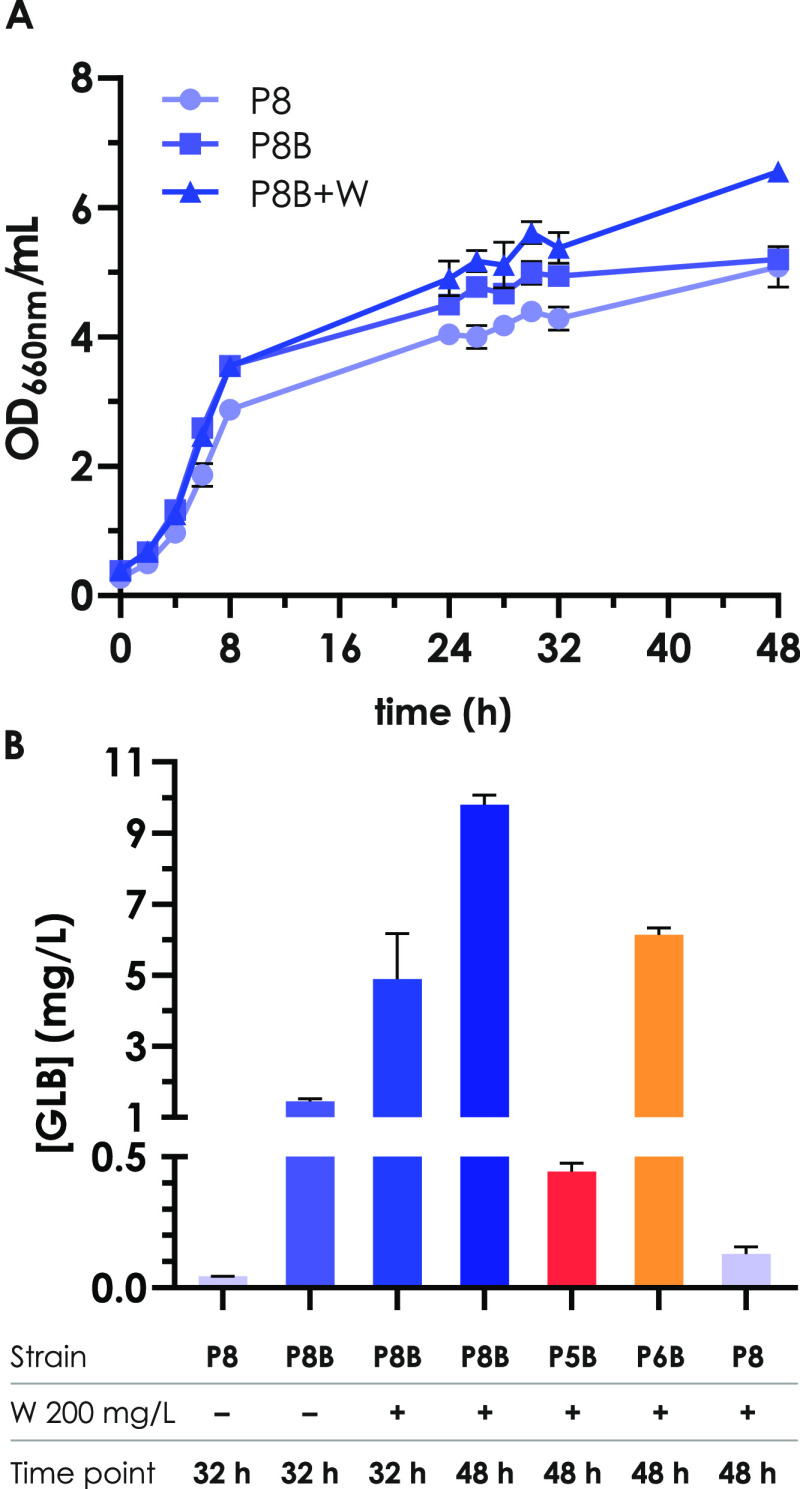
Growth curves and comparison of glucobrassicin production of different strains. (A) Growth curves of CER.P8, CER.P8.B, and CER.P8.B+W strains in shake flasks in minimal synthetic media. (B) Comparison of GLB production in the different engineered strains, in the presence or absence of tryptophan (±W 200 mg/L) at 32 or 48 h sampling time. P8: CER.P8; P8B: CER.P8.B; P5B: CER.P5.B; P6B: CER.P6.B.

At first, CER.P8 was grown in minimal synthetic media, samples were taken at the beginning of the stationary phase (32 h from the inoculum in the reported kinetics, Figure 4A) and analyzed by UHPLC-MS/MS. GLB titers resulted in 0.04 ± 0.001 mg/L (Figure 4B), while the literature described an *S. cerevisiae* strain able to reach 1.07 ± 0.381 mg/L GLB.^13^ Of note, we confirmed that all of the GLB produced was released in the media, while its residual intracellular amount was below the detection limit (data not shown). The main difference between the two strains is that in the previous study all of the coding sequences were under the control of the *GAL1*/*GAL10* inducible promoter. The higher titer obtained by Mikkelsen and colleagues can be explained by the fact that expression levels induced by *GAL* promoters are extremely high compared to *TPI1* and *PGK1* promoters used in this work.^14^ Considering further developments toward industrial exploitation, our setting was intended to be improved by not depending on galactose, which is an expensive carbon source.

Subsequently, we compared the GLB titer from the CER.P8 strain with CER.P8.B, and we obtained 0.04 ± 0.001 mg/L and 1.45 ± 0.072 mg/L, respectively (Figure 4B). The different titers suggested that the *Bo*CYP79B2 cytochrome presented a higher activity than *At*CYP79B2 when expressed in *S. cerevisiae* as the initiating enzyme for GLB production.

Regarding media composition, to further increase the flux toward GLB biosynthesis, we supplied cells with TRP as a direct precursor for the biosynthetic pathway. Indeed, many studies present in the literature show how the shikimate pathway is a rate-limiting step to obtain a wide variety of valuable products that can be derived from it.^21−23^ We provided yeast with tryptophan into the medium at the final concentration of 200 mg/L and analyzed the GLB production of the CER.P8.B strain, obtaining a titer of 4.89 ± 1.285 mg/L after 32 h (Figure 4B). This result confirmed our hypothesis and strongly suggested that GLB production is significantly determined by the first enzymatic reaction of the pathway and by precursor availability.

HPLC measurement showed that in our conditions at the beginning of the stationary phase ethanol is still present in the media, while it is completely depleted after 48 h from the inoculum. Coherently, we repeated the measurement of GLB production also at this time point and observed a concentration of 9.80 ± 0.267 mg/L (Figure 4B), almost the double of what was observed in an earlier sampling. To the best of our knowledge, this is the highest production of GLB ever obtained in recombinant cell factories and improved the results previously obtained by an order of magnitude.^13^ A second drawback of using *GAL1/GAL10* promoters is that once galactose is consumed *GAL* promoters stop working and the production too; in the present study, where constitutive promoters were used, we observed that the production increased over time, even after glucose depletion.

Furthermore, we compared the three different versions of the pathway present in the three strains CER.P5.B, CER.P6.B, and CER.P8.B (Figure 3B). For these experiments, samples were collected after carbon source exhausting (corresponding to 48 h from the inoculum in our experiments; Figure 4A) and GLB production was determined (Figure 4B).

Interestingly, the CER.P5.B+W strain produced 0.45 ± 0.040 mg/L, while the CER.P6.B+W strain produced a GLB amount 6 times higher, 6.14 ± 0.191 mg/L (Figure 4B). Since the two strains differed only in the expression of the ATR1 reductase, this underlined the importance of restoring the reduced state for the correct functionality of the cytochromes and for the enhancement of GLB production, as was shown in the previous work of Mikkelsen and colleagues.^13^ Interestingly, comparing the GLB production in CER.P6.B+W and CER.P8.B+W strains, it was possible to compare the cysteine-dependent pathway with the glutathione-dependent pathway. As shown previously, the highest amount of GLB was obtained with the strain CER.P8.B, which achieved a final titer of 9.80 ± 0.267 mg/L (Figure 4B). CER.P6.B+W reached a production of 6.14 ± 0.191 mg/L, highlighting that also the spontaneous reaction with cysteine could support a significant production of glucosinolates when the cytochrome functionality is further supported by the presence of ATR1; nonetheless, the glutathione-dependent pathway is a strategy to boost the production (Figure 4B).

#### “Fusion-Ready”: DBTL of Tagged Enzymes

The versatility of the toolkit in allowing the in-frame fusion of protein tags with the expressed enzymes was also tested. This third feature of the toolkit was applied to verify if all of the proteins were correctly translated and to identify possible different subcellular localizations of the expressed enzymes.

A new set of different strains was built, in which a GFP-tagged version of each of the eight genes encoding the enzymes of the pathway was singularly integrated in the same locus of the producing strains (see Table S1 and Figure S3 for details).

**Figure 5:**
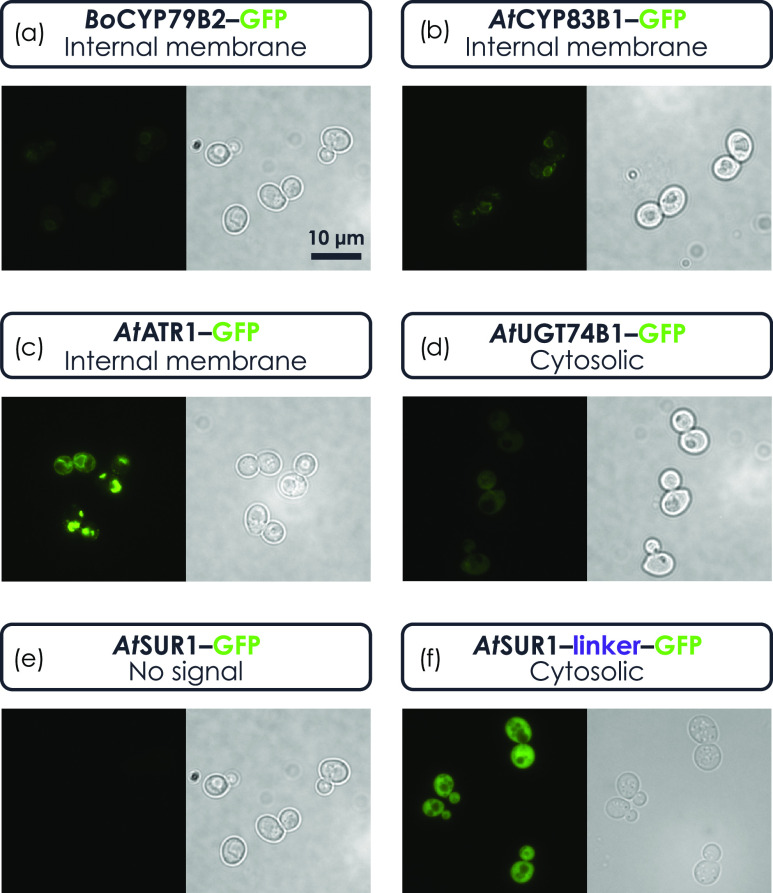
Representative pictures of strains expressing GFP-tagged enzymes with an indication of their putative subcellular localization. The “fuSion-ready” feature of the toolkit was applied to verify if all of the proteins were correctly translated and to identify possible different subcellular localizations of the expressed enzymes. For instance, *Bo*CYP79B2 (a), *At*CYP83B1 (b), and *At*ATR1 (c) show a pattern that can be attributed to localization in internal membranes, while *At*UGT74B1 (d) and *At*SUR1 (f) appear to be cytosolic. In order to obtain a detectable signal for *At*SUR1, a cycle of Design–Build–Test–Learn was carried out by inserting a protein linker between the enzyme and the GFP (e compared to f).

A fluorescence signal was visible for all of the GFP-tagged proteins, except for *At*SUR1-GFP (Figure 5E and Figure S3). Exploiting the modularity of the toolkit, a new construct with an in-frame form of GFP bearing a longer linker to connect it to *At*SUR1 was built, and the new strain showed the expected fluorescence (Figure 5F).

Figure 5 shows representative pictures of some of the GFP-tagged enzymes. It is possible to identify different patterns of the fluorescence signal, as an indication of different subcellular localizations. For instance, *Bo*CYP79B2, *At*CYP83B1, and *At*ATR1 show a pattern that can be attributed to localization in internal membranes (in comparison with the LoQAtE database; https://www.weizmann.ac.il/molgen/loqate/localization-categories), as it would be predicted for P450 cytochromes and the related reductase, while the other enzymes appear to be cytosolic. This shows how the “fuSion-ready” feature of the toolkit can be exploited to verify the correct localization expected for heterologous enzymes (e.g., predicted membrane proteins).

This confirms that the Easy-MISE toolkit allows for quick cycles of Design, Build, Test, and Learn. This rapidity is granted by its modular design and the simplicity of the single multiplasmid assembly, which reduces the time from part construction to the newly engineered strains.

## Conclusions

In conclusion, with this work, we remarked upon the importance of designing efficient, modular, and ready-to-use synthetic biology toolkits to accelerate the construction of the final desired cell factory and, overall, to improve yields, titers, and productivities of final strains. In recent years, there are several examples describing optimizations of already existing toolkits both for modular cloning^24^ and for genome editing.^25^

Here, we presented the Easy-MISE toolkit, and to prove its readiness and flexibility, we engineered *S. cerevisiae* to have a better understanding of how to optimize GLB production by exploiting different combinations of its biosynthetic pathway. Overall, the comparison of the data here presented, together with those of two previous works on the study of the GLB biosynthetic pathway, led to show that the best enzymatic combination for GLB production in *S. cerevisiae* comprises the glutathione-dependent pathway, the *Bo*CYP79B2 cytochrome, and the ATR1 reductase. The final GLB titer in the CER.P8.B strain grown in minimal glucose medium with 200 mg/L TRP was 9.80 ± 0.267 mg/L, 10-fold higher than the best result present in the literature.^13^ Although the current titers of GLB are far from the need for a commercial process, our work underlines that there is still great room for improvement to increase the final production.

As a possible implementation of the Easy-MISE toolkit, it is already undergoing the study of a synthetic protein scaffold with specific linkers to be integrated into the biomolecular toolkit. Indeed, we strongly believe that the combination of synthetic protein scaffolds with previously shown metabolic engineering techniques might allow for solving difficult biological issues.

In the end, this work shows the synergetic effect of combining synthetic biology tools and the test of different orthologues of the key pathway enzymes in accelerating the amelioration of cell factory performances.

## Methods

### Strains

The *S. cerevisiae* parental strain used in this study was CEN.PK 102-5B (*MATa*; *ura3-52*; *his3-11*; *leu2-3/112*; *TRP1*; *MAL2-8c*; *SUC2* – Dr. P. Kötter, Institute of Microbiology, Johann Wolfgang Goethe-University, Frankfurt, Germany).^26^ The parental strain was transformed with pYX series expression integrative vectors (R&D Systems, Inc.) to complement auxotrophies and obtain the CEN.PK*c* strain. All *S. cerevisiae* strains obtained in this work are described in the Results and Discussion section and listed in Table S1.

*E. coli* strain DH5α was used to clone, propagate, and store the plasmids.

### Media and Growth Conditions

*E. coli* strains were stored in cryotubes at −80 °C in 50% glycerol (v/v) and grown in Lysogeny broth (LB) medium (10 g/L NaCl, 10 g/L peptone, 5 g/L yeast extract) or Terrific broth (TB) media (20 g/L peptone, 24 g/L yeast extract, 4 mL/L glycerol, 0.17 M KH_2_PO_4_, 0.72 M K_2_HPO_4_). When needed, the medium was supplemented with 100 μg/mL ampicillin or 50 μg/mL kanamycin.

Yeasts were stored in cryotubes at −80 °C in 20% glycerol (v/v) and grown on YPD medium (20 g/L d-glucose, 20 g/L peptone, 10 g/L yeast extract) or YNB minimal synthetic medium (20 g/L d-glucose, 6,7 g/L YNB w/o amino acids (cat. no. 919-15, Difco Laboratories, Detroit, MI)). When needed, the medium was supplemented with antibiotics, G418 (500 mg/L) for the selection of the Cas9 plasmid, and/or nourseothricin (clonNAT) (100 mg/L) for the selection of the gRNA plasmid.

Agar plates were prepared as described above but with the addition of 20 g/L agar. Yeast extract was provided by Biolife Italiana S.r.l., Milan, Italy. All of the other reagents were provided by Sigma-Aldrich Co., St. Louis, MO.

For GLB production experiments, yeasts were grown in a minimal synthetic medium with or without the presence of tryptophan 200 mg/L. Yeast cells were pregrown until the exponential phase in the same medium, and the growth curves were obtained by inoculating at an initial optical density of 0.5 (660 nm). Optical density, sugar consumption, main secondary metabolite production, and GLB production were monitored at specific time intervals over 48 h from the inoculum. Each experiment was repeated at least three times. All strains were grown in shake flasks at 30 °C and on an Orbital shaker at 160 rpm, and the ratio of flask/medium volume was 5:1.

### Design of Plasmids and Constructs

All primers used in this work are listed in Table S2.

The pGA-blue plasmid, Figure S1, was obtained using pStBlue-1 as the backbone (Novagene) and the ORF encoding amilCP chromoprotein, a gift from Anthony Forster (Addgene plasmid # 117847; http://n2t.net/addgene:117847; RRID: Addgene 117847). Briefly, the pStBlue-1 backbone was amplified by PCR in two fragments with primers (i) bbKan_Fw and bbKan_Rv_inner and (ii) bbKan_Rv and bbKan_Fw_inner, to remove the *Bsa*I recognition site. The AmilCP chromoprotein ORF was amplified with two PCRs too, with (i) amilCP_Fw and amilCP_Rv_inner and (ii) amilCP_Rv and amilCP_Fw_inner, designed to remove an internal *Esp*3I recognition site. Once the four amplicons were obtained, a Golden Gate reaction was performed with the T4 ligase and *Bsa*I as the Type IIS restriction enzyme; indeed, the four primers mentioned have wings with the *Bsa*I recognition site in it (Figure S1).

pEM series plasmids were built cloning the sequence of interest, flanked by *Bsa*I and *Esp*3I recognition sites, into the pGA-blue plasmid as the destination plasmid and exploiting Golden Gate reactions with T4 ligase and *Esp*3I as the Type IIS restriction enzyme. Transformants were selected in the presence of kanamycin. PCR templates used to build the pEM library are listed in Table S3, as well as pEM plasmids’ names and primers used to PCR amplify each insert. All pEM plasmids were verified by colony PCRs performed with appropriate primers and then sequenced with the same primers thanks to Mix2Seq kit, Eurofins Genomics.

pGA-red plasmids, Figure S2, were obtained from YCplac33.^27^ First, the *Bsa*I recognition site was removed to obtain YCplac33_*Bsa*IFree. A fragment from YCplac33 was amplified by using *Bsa*IFree_Fw and *Bsa*IFree_Rv primers. We set up a Golden Gate reaction with the *Bsa*I restriction enzyme, YCplac33 and *Bsa*IFree_amplicon, creating YCplac33_*Bsa*IFree. The mRFP1 coding sequence was PCR amplified with RFP_acceptor_Fw and RFP_acceptor_Rv primers from the GGE114 plasmid, a gift from Macarena Larroude^28^ (Addgene plasmid #120731). YCplac33_*Bsa*IFree and the RFP amplicon were cut with *Bam*HI and *Sac*I and then ligated to create the pGA-red-maxi plasmid using the Quick Ligation Kit from New England Biolabs (NEB). To remove the *URA*3 region, a PCR was performed with pGA-red-maxi as the template and pGA-red-mini_Fw and pGA-red-mini_Rv as primers. The final amplicon was reclosed with a Golden Gate assembly reaction in the presence of *Esp*3I and the T4 ligase to obtain the pGA-red-mini plasmid.

Plasmids with integration constructs, level 1 plasmids, are listed in Table S4 and obtained by exploiting a Golden Gate reaction with the T4 ligase and *Bsa*I as Type IIS restriction enzymes. To generate level 1 plasmids, one of the two pGA-red plasmids was used as the destination plasmid and the library of pEM series plasmids was used as the donor. *E. coli* transformants were selected in the presence of ampicillin. All level 1 plasmids’ sequences were verified by PCRs.

The 5′ and 3′ protruding ends in pEM plasmids left by *Bsa*I during the Golden Gate reaction have been well-defined thanks to the work of Potapov and colleagues^17^ and are listed in Table 1.

Golden Gate assembly procedures followed in this work have been fully described in the dedicated section in the Supporting Information. All starting plasmids used in this work are listed in Table S5. Q5 High-Fidelity DNA Polymerase from NEB was used on a ProFlex PCR System (Life Technologies) following the NEB manual. All enzymes utilized are from NEB.

### Parts of the Easy-MISE Toolkit

Promoters present in pEM plasmids were selected thanks to the work of Peng and colleagues,^14^ and the two terminators are the same as used by Mikkelsen and colleagues.^13^

Sequences for open reading frames of the GLB pathways *AtCYP*79*B*2, *AtCYP*83*B*1, *AtSUR*1, *AtUGT*74*B*1, *AtSOT*16, *AtGSTF*9, *AtGGP*1, and *AtATR*1 were obtained from *A. thaliana* protein sequences registered in The Arabidopsis Information Resource (TAIR),^29^ translated into DNA sequences codon-optimized for *S. cerevisiae* and synthesized by Twist Bioscience. All synthetic sequences used in this work are listed in Table S7. *BoCYP*79*B*2 from *B. oleracea* var. *botrytis* was PCR amplified from p012bT[CYP83][CYP79].^12^

### Yeast Transformation

Yeast transformants were obtained by exploiting the EasyClone-MarkerFree toolkit^7^ and the constructs created in this work.

The gRNA helper vectors (natMX as the dominant marker) and the Cas9 plasmid pCfB2312 (kanMX as the dominant marker) come from the EasyClone-MarkerFree vector set, a gift from Irina Borodina (Addgene kit #1000000098). All of the transformations were performed following the EasyClone-MarkerFree manual. The starting yeast carrying the Cas9 plasmid (pCfB2312) was obtained by adding to the transformation mix 500 ng of the Cas9 expression vector and selecting transformants onto YPD+G418 media. Plasmids with integration constructs were linearized with *Nhe*I, and the integration fragments were gel-purified and transformed (500 ng) along with a gRNA helper vector (500 ng) into yeasts already carrying the Cas9 plasmid (pCfB2312). Correct integration of the vectors into the genome was verified by colony PCR using primers listed in Table S2 and named “ctr_integr”.

Once positive clones were obtained and verified, the gRNA helper vector was removed by optimizing the curing protocol: a single colony was inoculated in 5 mL of YPD + G418 at 30 °C, 160 rpm overnight. Then, about 100 cells were plated on a YPD + G418 plate and incubated at 30 °C for 2 days. To verify the gRNA helper vector loss, single colonies were grown overnight in two different media: YPD with G418 and YPD with clonNAT; cells without a gRNA helper vector will not be able to grow on media with clonNAT. If the Cas9 expression vector needs to be removed too, the procedure is the same, but the single colony is grown o/n and plated on YPD agar plates with no selection, and in the last step, single colonies are grown in YPD media with no selection too: cells without the gRNA helper vector and the Cas9 expression vector will not be able to grow on media with antibiotics.

### Colony PCRs

To perform colony PCRs, at least five different *E. coli* colonies were picked for each transformation plate and dissolved (i) in 20 μL of growth media with the proper antibiotic as a colony backup and (ii) into the PCR tube with the appropriate PCR mix. To boost cell disruption, the initial denaturation step must last at least 5 min. The positive *E. coli* clones are then inoculated starting from the 20 μL liquid cultures prepared at the beginning.

To perform colony PCRs on *S. cerevisiae* colonies, genomic DNA was extracted following the LiOAc-SDS optimized procedure of Lõoke et al.^30^ After obtaining the genomic DNA, 1 μL of the supernatant was used as the PCR template. The positive clones were then inoculated in the correct growth media.

Wonder Taq DNA polymerase (Euroclone) was used on a ProFlex PCR System (Life Technologies) to perform colony PCR reactions.

### Fluorescence Microscopy Analysis

Yeast cells were grown in minimal synthetic medium and harvested in the exponential phase. 1 mL of the culture was collected and centrifuged at 6000 rpm for 5 min, and the pellet was resuspended in phosphate-buffered saline solution (PBS; NaH_2_PO_4_ 53 mM, Na_2_HPO_4_ 613 mM, NaCl 75 mM). Cells were then observed with a Nikon Eclipse 90i fluorescence microscope (Nikon) equipped with a ×100 objective. Images were acquired with a Digital Sight DS-U3 Nikon camera using NIS-Elements software (version 4.3). GFP-tagged proteins were observed using the B-2A (EX 450-490 DM 505 BA 520) filter (Nikon). Digital images were acquired with a CoolSnap CCD camera (Photometrics), using MetaMorph 6.3 software (Molecular Devices).

### Quantitative Analysis of GLB by UHPLC-MS/MS

The quantitative analysis of GLB was performed on a Shimadzu Nexera UHPLC system (Shimadzu, Milano, Italy), consisting of a CBM-20A controller, two LC-30AD dual-plunger parallel-flow pumps, a DGU-20A5 degasser, a CTO-20A column oven, and a SIL-30AC autosampler. The UHPLC system was interfaced with an API-6500 triple quadrupole mass spectrometer (AB Sciex, Toronto, Canada) equipped with a TurboIonSpray source operating in the negative ion mode for the detection of the analyte. The samples were chromatographed on a Kinetex C18, UHPLC column (100 × 2.1 mm^2^, 2.7 μm; Phenomenex, Bologna, Italy), using H_2_O (A) and CH_3_CN (B), both with 0.1% HCOOH as mobile phases. After injection (10 μL), the analyte was eluted using the following gradient: 0–1 min, 5% B, 1–3 min, linear increase to 50% B hold of 1.0 min, 4–5 min, linear increase to 80% B, 5–7, linear increase from 80 to 95% B. The column was kept at 30 °C and the flow rate was set at 0.4 mL/min for all of the chromatographic runs. At the end of each run, the column was washed with 95% B to remove the matrix interferents and re-equilibrated with 5% B for 4 and 5 min, respectively. Analyst software version 1.6 (AB Sciex, Toronto, Canada) was used for mass spectrometer control and data acquisition/processing.

To improve the analyte ionization and to select the multiple reaction monitoring (MRM) transitions, tune optimization was carried out by the direct infusion of GLB standard solution at a concentration of 5 μg/mL. The optimized ion source parameters were as follows: ion spray voltage (IS) −4500 V, source temperature (TEM) 400 °C, dwell time was 20 ms for each MRM transition, nebulizer gas (GS1) 40 psi, heater gas (GS2) 40 psi, curtain gas (CUR) 30 psi, collision gas (CAD) medium. Nitrogen was used for both nebulizer and collision gas, and collision energies were optimized for each analyte transition during infusion of the pure standard. For the proposed method, the most intense transitions and one characteristic ion were chosen for quantification and confirmation of the analyte, respectively. In particular, for analyte quantification, the selected MS/MS transition of GLB was *m*/*z* 447.0  → 96.0 (CE = −30), whereas for analyte identification *m*/*z* 447.0 → 259.0 (CE = −40) was used. For quantitative determination of the target compound, a stock solution (1 mg/mL) of GLB was used as an external standard (ES). The calibration curve was obtained by plotting the GLB peak area versus concentration (mg/mL) and by diluting the appropriate volume of stock solution in H_2_O/CH_3_CN (8:2 v/v). The calibration curve was evaluated at six levels in the range of 0.1–12 μg/mL and an ANOVA test was performed to check linearity (*R*^2^ = 0.9989).

To analyze the extracellular concentration of GLB, 1 mL of cell culture was centrifuged at 13 000 rpm for 10 min. The supernatants were diluted (when appropriate) in milliQ water, and the concentrations were determined by UHPLC-MS/MS analysis. To quantify total GLB production, 1 mL of each cell culture was transferred to 2 mL FastPrep tubes containing 0.2 mL of acid-washed glass beads (0.45–0.55 mm). The FastPrep tubes were processed 3 times for 20 s in a FastPrep FP120 Instrument (Savant Instruments, New York). After centrifugation at 13 000 rpm for 10 min, the supernatant was injected into the system. The intracellular GLB content was deducted by subtraction between the total amount of GLB and the extracellular fraction.

## Supporting Information Available

Golden Gate assembly protocol, strain construction; Figure S1, construction of pGA-blue plasmid; Figure S2, construction of pGA-red plasmid; Figure S3, fluorescence microscopy of GFP-tagged enzymes confirming their expression in *S. cerevisiae*; Table S1, yeast strains; Table S2, primers; Table S3, pEM series plasmids; Table S4, level 1 plasmids; Table S5, other plasmids used in this work; Table S6, integration efficiencies for each Easy-MISE toolkit genome loci; Table S7, synthetic sequences (PDF)
