# Supplementary material for: Easy Modular Integrative fuSion-ready Expression (Easy-MISE) Toolkit for Fast Engineering of Heterologous Productions in Saccharomyces cerevisiae
Source: ACS Synth Biol. 2023 Apr 14;12(5):1508–19. doi: 10.1021/acssynbio.3c00015 (PMC10204092; doi:10.1021/acssynbio.3c00015)
Supplement: Supplementary file 1 — sb3c00015_si_001.pdf [file sb3c00015_si_001.pdf]

Supplementary information

An Easy Modular Integrative fuSion-ready Expression (Easy-MISE) toolkit for fast engineering of heterologous productions in *Saccharomyces cerevisiae*

Letizia Maestroni[†], Pietro Butti[†], Riccardo Milanesi, Stefania Pagliari, Luca Campone, Immacolata Serra, Paola Branduardi[*]

[*]Corresponding author: paola.branduardi@unimib.it

[†]Letizia Maestroni and Pietro Butti contributed equally to this work

[1]Department of Biotechnology and Biosciences, University of Milano Bicocca, Piazza della Scienza 2, 20126 Milan, Italy

TABLE OF CONTENTS

Supplementary methods 3

- Golden Gate assembly protocol 3
- Strain construction 4

Supplementary figures 5

- Figure S1: Construction of pGA-blue plasmid 5
- Figure S2: Construction of pGA-red plasmid 6
- Figure S3: Fluorescence microscopy of GFP-tagged enzymes confirms their expression in *S. cerevisiae* 7

Supplementary tables 8

- Table S1: Yeast strains 8
- Table S2: Primers 9
- Table S.3: pEM series plasmids 12
- Table S.4: Level 1 plasmids 14
- Table S.5: Other plasmids used in this work 15
- Table S.6: Integration efficiencies for each Easy-MISE toolkit genome loci 15
- Table S.7: Synthetic sequences 16

SUPPLEMENTARY METHODS

Golden Gate assembly protocol

1. Set up 20 µl assembly reactions as follows:

REAGENTS	< 10 fragments	> 10 fragments
Destination Plasmid	0.025 pmol	0.05 pmol
Inserts (user provided):<br>- if precloned<br>- if in amplicon form	 75 ng each plasmid<br>0.10 pmol each amplicon	 75 ng each plasmid<br>0.10 pmol each amplicon
T4 DNA Ligase Buffer (10X)	2.0 µL	2.0 µL
T4 DNA Ligase, 400 U/µl	0.25 µL (100 units)	0.5 µL (200 units)
BsaI-HFv2, 20 U/µl **or** Esp3I, 10 U/µl	12.5 units	25 units
Nuclease-free H_2O	up to 20 µL	up to 20 µL

2. Mix gently by pipetting.
3. Transfer to thermocycler and use one of the following programs:

   1-5 inserts (e.g. pEMs construction): (37°C, 2 min → 16°C, 2 min) x 30 → 60°C, 5 min

   6+ inserts (e.g. Easy-MISE final assembly): (37°C, 5 min → 16°C, 5 min) x 30 → 60°C, 5 min

If reactions are done overnight, add a 4 °C terminal hold to the protocol, but repeat the final 5 min 60 °C step the next day before the transformations.

4. At the end, transform *E. coli*. For quantities, please refer to these guidelines:

   1-5 inserts (e.g. pEMs construction): 2 µL of the mix and plate 1:5 of cellular suspension

   6+ inserts (e.g. Easy-MISE final assembly): 5 µL of the mix and plate everything

Strain construction

We transformed CEN.PK*c* strain with pCfB2312 plasmid, containing Cas9 coding sequence under the control of p*TEF1* promoter and harbouring the kanMX cassette as a dominant marker. The CEN.PKc+Cas9 strain was then transformed simultaneously with G5, G6 and G7 cassettes (Table S4) together with plasmid pCfB3051, carrying three gRNAs to concurrently target X-3, XI-2 and XII-2 loci and natMX as dominant marker, obtaining a strain expressing the 5-gene version of the pathway, called CER.P5. This strain, containing *AtCYP79B2*, *AtCYP83B1*, *AtSUR1*, *AtUGT74B1* and *AtSOT16* integrated into the genome, was then transformed with G11 cassette (Table S4), containing *AtATR1*, together with plasmid pCfB3045, carrying gRNA to XI-3 locus and natMX, to obtain CER.P6. The last transformation step was carried out using G8, containing *AtGSTF9* and *AtGGP1* as integration cassettes (Table S4) and the plasmid pCfB3042, carrying gRNA to X-4 locus and natMX. This strain was called CER.P8. CER.P8.B strain (expressing cytochrome CYP79B2 from *B. oleracea var. botrytis*) was obtained following the same workflow presented for CER.P8, therefore we previously obtained CER.P5.B strain and then the CER.P6.B strain, with the contribution of *At*ATR1 reductase. In Table S1 all the genotypes are reported and Figure 3B shows a schematic representation of the final strains obtained in this work.

We transformed CEN.PKc+Cas9 respectively with G22 (*AtCYP83B1* tagged with GFP) and pCfB3041 (carrying gRNA to X-3 locus and natMX), G23 (*AtGSTF9* tagged with GFP) and pCfB3042 (carrying gRNA to X-4 locus and natMX), G24 (*AtGGP1* tagged with GFP) and pCfB3042 (carrying gRNA to X-4 locus and natMX), G25 (*AtSUR1* tagged with GFP) and pCfB3044 (carrying gRNA to XI-2 locus and natMX), G26 (*AtUGT74B1* tagged with GFP) and pCfB3044 (carrying gRNA to XI-2 locus and natMX), G10 (*AtSOT16* tagged with GFP) and pCfB3048 (carrying gRNA to XII-2 locus and natMX), G27 (*AtATR1* tagged with GFP) and pCfB3045 (carrying gRNA to XI-3 locus and natMX). All strain genotypes are listed in Table S1.

SUPPLEMENTARY FIGURES

Figure S1: Construction of pGA-blue plasmid

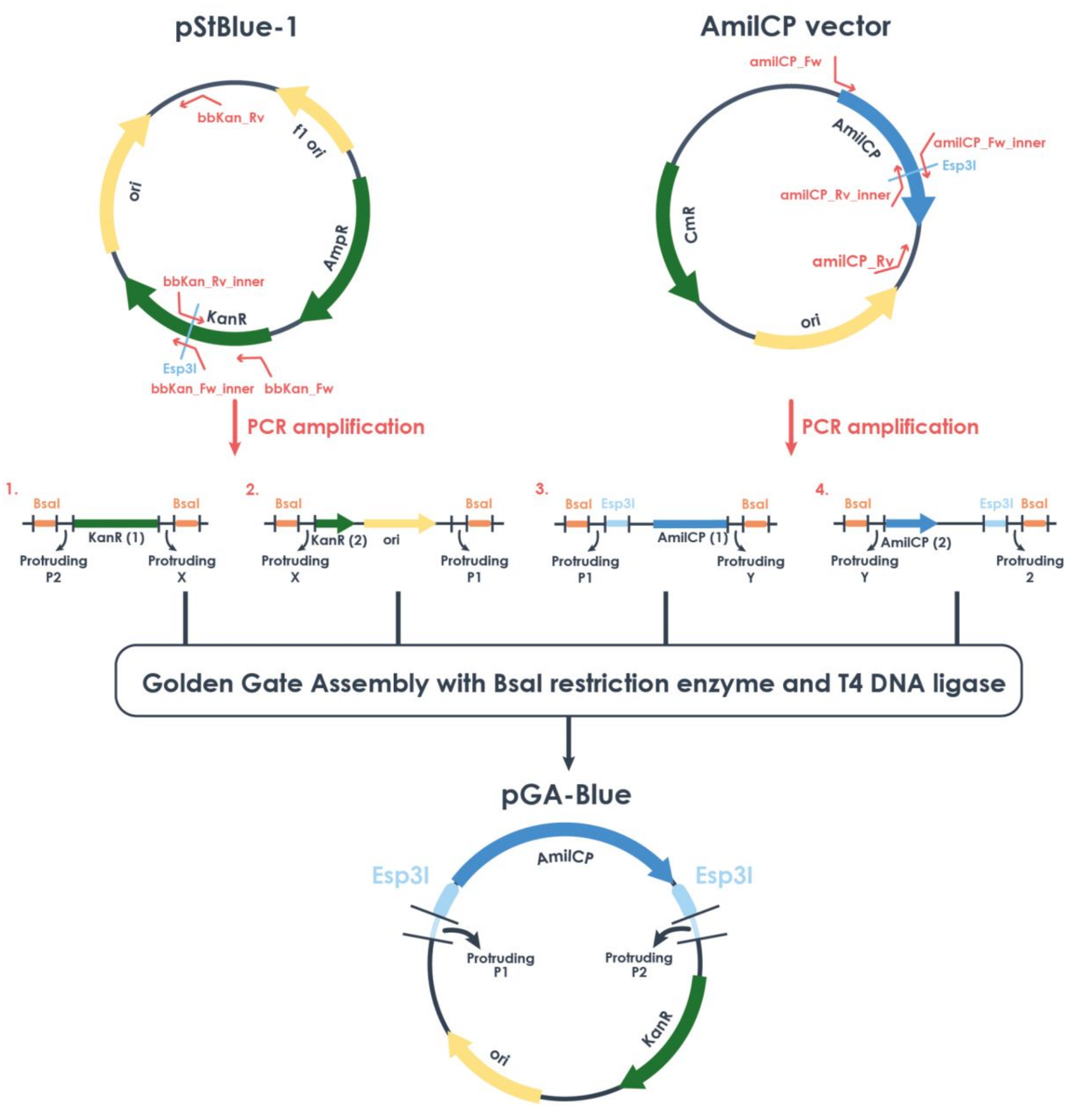

pGA-blue plasmid was built starting from pStBlue-1 plasmid and an *E. coli* vector for the expression of the AmilCP chromoprotein. In particular, pGA-blue backbone, containing *kanR* and Ori sequences, has been amplified from pStBlue-1 with primers added with flanking sequences carrying BsaI restriction sites and compatible overhangs. Since *kanR* sequence contains an Esp3I restriction site, two inner primers have been used to amplify *kanR* and mutate Esp3I binding site. This led to the amplification of the backbone in two parts (part 1 and part 2) subsequently cloned in pGA-blue. Similarly, AmilCP coding sequence has been amplified in two parts (part 3 and part 4) to remove an Esp3I restriction site, while its 5' and 3' ends have also been added with Esp3I restriction sites.

The four obtained parts have been used as substrates of a Golden Gate reaction carried out with BsaI and resulting in the construction of the pGA-blue plasmid. The assembly product has been transformed into DH5α for amplification. One blue colony has been checked with colony PCR and further confirmed by sequencing.

Figure S2: Construction of pGA-red plasmid

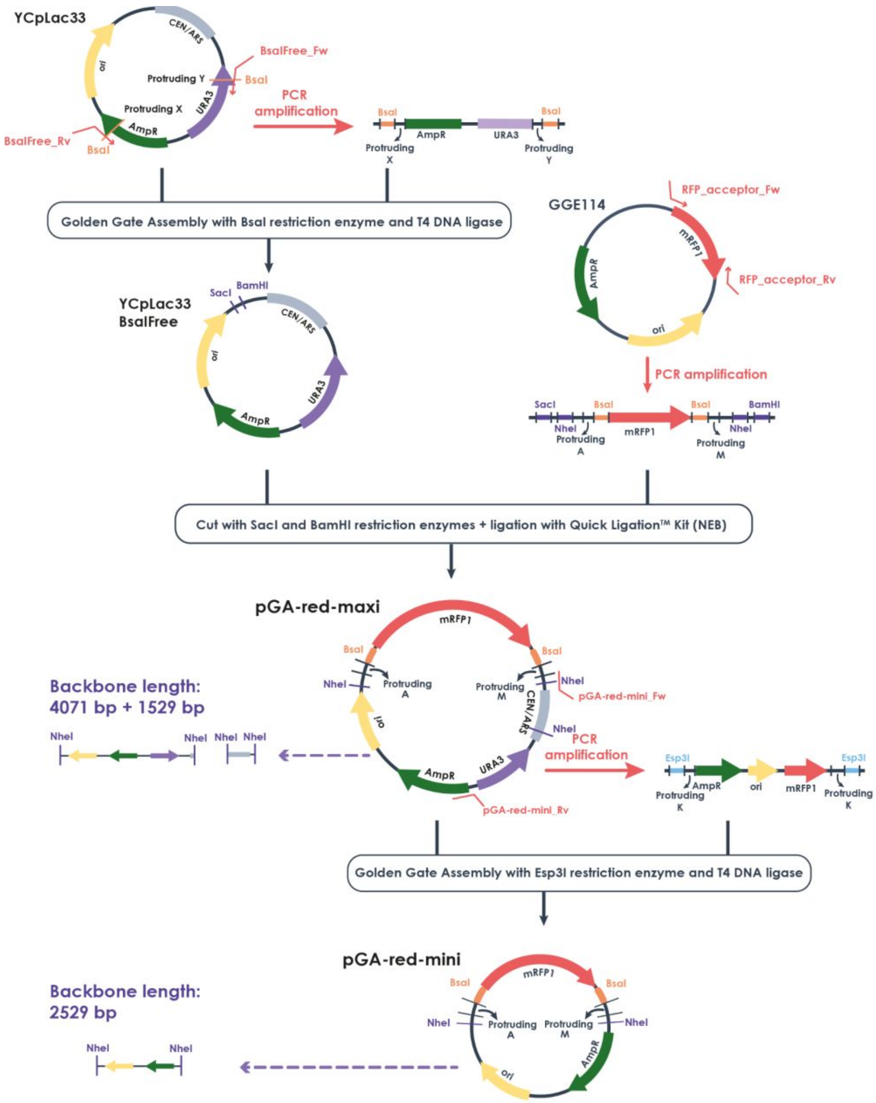

pGA-red-maxi and pGA-red-mini plasmids were built obtaining the backbone and the chromoprotein from YCpLac33 and GGE114, respectively. As regards the backbone, *ampR* and *URA3* markers were amplified from YCpLac33 with primers added with flanking sequencing containing BsaI restriction sites and designed to mutate the native BsaI sites. YCpLac33-BsaIFree was obtained with a Golden Gate Assembly reaction and exploiting YCpLac33 native BsaI sites to replace native *ampR* and *URA3* with a BsaI free version. The pGA-red-maxi plasmid was obtained by amplifying mRFP1 from GGE114 with primers added with BsaI, SacI and BamHI restriction sites. mRFP1 was cloned in the YCpLac33-BsaIFree backbone, digesting both the amplicon and the plasmid with SacI and BamHI and carrying out the ligation with the Quick Ligation Kit (NEB). Of note, the primers used for mRFP1 amplification were designed to introduce A and M protruding bases and NheI restriction sites that will be exploited for the construction of the G series plasmids. pGA-red-mini plasmid was obtained starting from pGA-red-maxi removing *URA3* and *CEN/ARS* sequences. This was achieved by amplifying pGA-red-maxi with primers designed to anneal upstream *URA3* and downstream *CEN/ARS* and added with flanking sequences carrying Esp3I restriction sites and compatible overhangs. The circularization of the reduced backbone was obtained by exploiting the compatible overhangs in a Golden Gate Assembly reaction carried out with Esp3I.

Figure S3: Fluorescence microscopy of GFP-tagged enzymes confirms their expression in *S. cerevisiae*

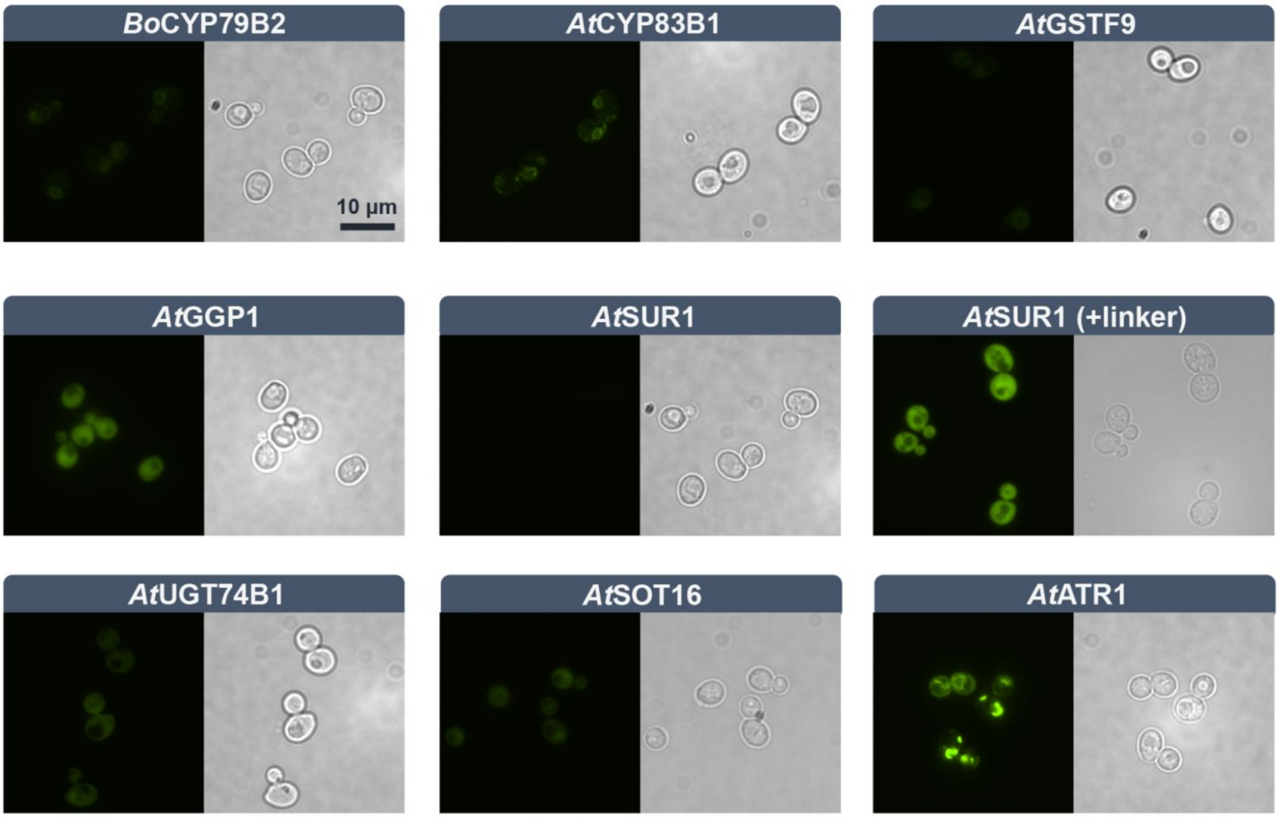

Protein expression of glucobrassicin biosynthetic enzymes of the final producing strain was verified by building yeast strains expressing GFP-tagged versions of the proteins. The tagged enzymes are integrated into the same locus and with the same promoters used in the producing strains. Fluorescence microscopy shows that all the enzymes were detectable, except for *At*SUR1. The latter was observed by adding a linker before the GFP tag. All the pictures were acquired under the same conditions.

SUPPLEMENTARY TABLES

Table S1: Yeast strains

Strain name	Parental strain	Genotype	
CEN.PK 102-5B		*MATa*, *ura3-52*, *his3-11*, *leu2-3/112*, *TRP1*, *MAL2-8c*, *SUC2*	
CEN.PK *C*	CEN.PK 102-5B	*MATa*, *ura3-52::URA3*, *his3-11::HIS3*, *leu2-3/112::LEU2*, *TRP1*, *MAL2-8c*, *SUC2*	
CER.P5	CEN.PK *C*	CEN.PK *C*	X-3::tADH1-**C79At**-pTPI1-pPGK1-**C83At**-tCYC1, XI-2::tADH1-**SURAt**-pTPI1-pPGK1-**UGTAt**-tCYC1,<br>XII-2::tADH1-**SOTAt**-pTPI1
CER.P6	CER.P5	CEN.PK *C*	X-3::tADH1-**C79At**-pTPI1-pPGK1-**C83At**-tCYC1, XI-2::tADH1-**SURAt**-pTPI1-pPGK1-**UGTAt**-tCYC1,<br>XII-2::tADH1-**SOTAt**-pTPI1<br>XI-3::pPGK1-**ATRAt**-tCYC1
CER.P8	CER.P6	CEN.PK *C*	X-3::tADH1-**C79At**-pTPI1-pPGK1-**C83At**-tCYC1,<br>XI-2::tADH1-**SURAt**-pTPI1-pPGK1-**UGTAt**-tCYC1,<br>XII-2::tADH1-**SOTAt**-pTPI1,<br>XI-3::pPGK1-**ATRAt**-tCYC1,<br>X-4::tADH1-**GSTAt**-pTPI1-pPGK1-**GGPAt**-tCYC1
CER.P5.B	CEN.PK *C*	CEN.PK *C*	X-3::tADH1-**C79Bo**-pTPI1-pPGK1-**C83At**-tCYC1, XI-2::tADH1-**SURAt**-pTPI1-pPGK1-**UGTAt**-tCYC1,<br>XII-2::tADH1-**SOTAt**-pTPI1
CER.P6.B	CER.P5B	CEN.PK *C*	X-3::tADH1-**C79Bo**-pTPI1-pPGK1-**C83At**-tCYC1, XI-2::tADH1-**SURAt**-pTPI1-pPGK1-**UGTAt**-tCYC1,<br>XII-2::tADH1-**SOTAt**-pTPI1,<br>XI-3::pPGK1-**ATRAt**-tCYC1
CER.P8.B	CER.P6B	CEN.PK *C*	X-3::tADH1-**C79Bo**-pTPI1-pPGK1-**C83At**-tCYC1, XI-2::tADH1-**SURAt**-pTPI1-pPGK1-**UGTAt**-tCYC1,<br>XII-2::tADH1-**SOTAt**-pTPI1,<br>XI-3::pPGK1-**ATRAt**-tCYC1,<br>X-4::tADH1-**GSTAt**-pTPI1-pPGK1-**GGPAt**-tCYC1
G22	CEN.PK *C*	CEN.PK *C*	X-3::pPGK1-**C83At-GFP**-tCYC1
G23	CEN.PK *C*	CEN.PK *C*	X-4::tADH1-**GFP-GSTAt**-pTPI1
G24	CEN.PK *C*	CEN.PK *C*	X-4::pPGK1-**GGPAt-GFP**-tCYC1
G25	CEN.PK *C*	CEN.PK *C*	XI-2::tADH1-**GFP-SURAt**-pTPI1
G26	CEN.PK *C*	CEN.PK *C*	XI-2::pPGK1-**UGTAt-GFP**-tCYC1
G10	CEN.PK *C*	CEN.PK *C*	XII-2::tADH1-**GFP-SOTAt**-pTPI1
G27	CEN.PK *C*	CEN.PK *C*	XI-3:: pPGK1-**ATRAt-GFP**-tCYC1
G43	CEN.PK *C*	CEN.PK *C*	XI-2::tADH1-**GFP+linker-SURAt**-pTPI1
G31	CEN.PK *C*	CEN.PK *C*	X-3::tADH1-**GFP-C79Bo**-pTPI1

Table S2: Primers

Name	Sequence
bbKan_Fw	TGCCAAGGTCTCCGACCCTGTCAGACCAAGTTTACTC
bbKan_Rv_inner	TATCGCGGTCTCTTGAGCGAGTCGAAATACG
bbKan_Rv	TCGATCGGTCTCCACCAGAATCGGCCAACGCGC
bbKan_Fw_inner	TGCCAAGGTCTCTCTCAGGCGCAATC
amilCP_Fw	TGCCAAGGTCTCCTGGTAGAGACGTTTACGGCTAGCTCAGTCC
amilCP_Rv_inner	TATCGCGGTCTCTGCAAAGAGTCGCTCAGTG
amilCP_Rv	TCGATCGGTCTCCGGTCAGAGACGCTGCATAACGCGAAGTAATC
amilCP_Fw_inner	TGCCAAGGTCTCTTTGCACGAG
BsaIFree_Fw	GGCTACGGTCTCCCTGTAGAGACTACATCATCCACGGTTC
BsaIFree_Rv	GGCTACGGTCTCCACCGCGAGATCCACGCTCACCG
RFP_acceptor_Fw	TCGATCGGATCCGCTAGCTGCCAGAGACCCGCAATTAATGTGAGTTAGCTCACTC
RFP_acceptor_Rv	TGCCAAGAGCTCGCTAGCTTGCAGAGACCTATATAAACGCAGAAAGGCCCACCCG
pGA-red-mini_Fw	GGAAGCGTCTCAAAAAAATGGTTTCTTAGACGTCAG
pGA-red-mini_Rv	GGAAGCGTCTCATTTTCCGATATATGGACTTCCAC
Seq_pGA-Red_Fw	CATGATTACGCCAAGCTTGC
Seq_pGA-Red_Rv	GGTAACGCCAGGGTTTTCCC
Seq_pGA-Blue_Fw	CCATGATTACGCCAAGCTCT
Seq_pGA-Blue_Rv	CCATTTAGGTGACACTATAG
pTDH3_EF_Fw	TGCCAACGTCTCATGGTCTCCGAGCTTTGTTTGTTTATGTGTGTTTATTC
pTDH3_EF_Rv	TCGATCCGTCTCAGGTCTCTTCCTTCCTTGATTACGTAAGGGAG
pTDH3_FG_Fw	TGCCAACGTCTCATGGTCTCCAGGATCCTTGATTACGTAAGGGAG
pTDH3_FG_Rv	TCGATCCGTCTCAGGTCTCTGAATTTTGTTTGTTTATGTGTGTTTATTC
pENO2_EF_Fw	TGCCAACGTCTCATGGTCTCCGAGCTATTATTGTATGTTATAGTATTAGTTGC
pENO2_EF_Rv	TCGATCCGTCTCAGGTCTCTTCCTCGGAAGTGTCTCATAAACTTTAC
pENO2_FG_Fw	TGCCAACGTCTCATGGTCTCCAGGACGGAAGTGTCTCATAAACTTTAC
pENO2_FG_Rv	TCGATCCGTCTCAGGTCTCTGAATTATTATTGTATGTTATAGTATTAGTTGC
pPGK1_EF_Fw	TGCCAACGTCTCATGGTCTCCGAGCTGTTTTATATTTGTTGTAAAAAGTAG
pPGK1_EF_Rv	TCGATCCGTCTCAGGTCTCTTCCTCCTCATACTATTATCAGGGC
pPGK1_FG_Fw	TGCCAACGTCTCATGGTCTCCAGGACCTCATACTATTATCAGGGC
pPGK1_FG_Rv	TCGATCCGTCTCAGGTCTCTGAATTGTTTTATATTTGTTGTAAAAAGTAG
pTPI1_EF_Fw	TGCCAACGTCTCATGGTCTCCGAGCTTTTTAGTTTATGTATGTGTTTTTTG
pTPI1_EF_Rv	TCGATCCGTCTCAGGTCTCTTCCTTGTTTAAAGATTACGGATATTTAAC
pTPI1_FG_Fw	TGCCAACGTCTCATGGTCTCCAGGATGTTTAAAGATTACGGATATTTAAC
pTPI1_FG_Rv	TCGATCCGTCTCAGGTCTCTGAATTTTTTAGTTTATGTATGTGTTTTTTG
pCYC1_EF_Fw	TGCCAACGTCTCATGGTCTCCGAGCTTAAGTCGTTTCTGTCTTTTTCCTTC
pCYC1_EF_Rv	TCGATCCGTCTCAGGTCTCTTCCTTCATTTGGCGAGCGTTGG
pCYC1_FG_Fw	TGCCAACGTCTCATGGTCTCCAGGATCATTTGGCGAGCGTTGG
pCYC1_FG_Rv	TCGATCCGTCTCAGGTCTCTGAATATTAAGTCGTTTCTGTCTTTTTC
pPDA1_EF_Fw	TGCCAACGTCTCATGGTCTCCGAGCTGGCACAAATGTGGTTTC
pPDA1_EF_Rv	TCGATCCGTCTCAGGTCTCTTCCTGAAATTCAAAACTCTCCAGAC
pPDA1_FG_Fw	TGCCAACGTCTCATGGTCTCCAGGAGAAATTCAAAACTCTCCAGAC
pPDA1_FG_Rv	TCGATCCGTCTCAGGTCTCTGAATTGGCACAAATGTGGTTTC
tADH1_BC_Fw	TGCCAACGTCTCATGGTCTCCACTAGAGCGACCTCATGCTATACC
tADH1_BC_Rv	TCGATCCGTCTCAGGTCTCTTCTGGCGAATTTCTTATGATTTATG
tCYC1_IL_Fw	TGCCAACGTCTCATGGTCTCCATAGATCCGCTCTAACCGAAAAGG
tCYC1_IL_Rv	TCGATCCGTCTCAGGTCTCTGTAACTTCGAGCGTCCCAAAACC
Adap_CD_Fw	TGCCAACGTCTCATGGTCTCCCAGAAGGGAGCTTCCAGGGGGAAAC
Adap_CD_Rv	TCGATCCGTCTCAGGTCTCTAGTTCTTGATAAGCCCCCCTGACGAG

Adap_HI_Fw	TGCCAACGTCTCATGGTCTCCACCGGTTGATAACCCAGCTTGG
Adap_HI_Rv	TCGATCCGTCTCAGGTCTCTCTATGATACCTGTCCGCCTTTC
Adap_BF_Fw	TGCCAACGTCTCATGGTCTCCACTATTGAAAAGCTGTGGTATGG
Adap_BF_Rv	TCGATCCGTCTCAGGTCTCTTCCTAGACGGTCACAGCTTGTC
Adap_FL_Fw	TGCCAACGTCTCATGGTCTCCAGGATTGAAAAGCTGTGGTATGG
Adap_FL_Rv	TCGATCCGTCTCAGGTCTCTGTAAAGACGGTCACAGCTTGTC
GFP_CD_Fw	TGCCAACGTCTCATGGTCTCCCAGATTATTTGTATAGTTCATCCATG
GFP_CD_Rv	TCGATCCGTCTCAGGTCTCTAGTTCTAGTAAAGGAGAAGAACTTTTC
GFP_HI_Fw	TGCCAACGTCTCATGGTCTCCACCGGTAGTAAAGGAGAAGAACTTTTC
GFP_HI_Rv	TCGATCCGTCTCAGGTCTCTCTATTTATTTGTATAGTTCATCCATG
GFP+linker_CD_Rv	TCGATCCGTCTCAGGTCTCTAGTTCTGGTTCTGGTGGCTC
GFP+linker_HI_Fw	TGCCAACGTCTCATGGTCTCCACCGGTGGTTCTGGTGGCTC
mCh_CD_Fw	TGCCAACGTCTCATGGTCTCCCAGACTACTTGTACAGCTCGTCCATG
mCh_CD_Rv	TCGATCCGTCTCAGGTCTCTAGTTCTGTGAGCAAGGGCGAGG
mCh_HI_Fw	TGCCAACGTCTCATGGTCTCCACCGGTGTGAGCAAGGGCGAGG
mCh_HI_Rv	TCGATCCGTCTCAGGTCTCTCTATCTACTTGTACAGCTCGTCCATG
X3_UP_AB_Fw	TGCCAACGTCTCATGGTCTCCTGCCAGAACGAGATCTTTGTGTTCG
X3_UP_AB_Rv	TCGATCCGTCTCAGGTCTCTTAGTTCGCCTACTTCTTGCCTATTG
X3_DW_LM_Fw	TGCCAACGTCTCATGGTCTCCTTACTAGTAAAAGGACGAGCTCAAGAG
X3_DW_LM_Rv_mut	TGCCAACGTCTCCGTCAAGACACGGGTATAAG
X3_DW_LM_Fw_mut	TATACCCGTCTCTTGACGC
X3_DW_LM_Rv	TCGATCCGTCTCAGGTCTCTTTGCGGTTTCCTGAAGGAAAAAGAGG
XI2_UP_AB_Fw	TGCCAACGTCTCATGGTCTCCTGCCGGAGCAGGATGAGGAGAAATAG
XI2_UP_AB_Rv	TCGATCCGTCTCAGGTCTCTTAGTGCCTAGTCTTGTTCTATGGCAC
XI2_DW_LM_Fw	TGCCAACGTCTCATGGTCTCCTTACGCGCTTTCAGATATTAAAAAGTTTAG
XI2_DW_LM_Rv	TCGATCCGTCTCAGGTCTCTTTGCGGGAAGATTCCGCTCTAC
XII2_UP_AB_Fw	TGCCAACGTCTCATGGTCTCCTGCCAATCAAAATCCCATATGTGACGC
XII2_UP_AB_Rv	TCGATCCGTCTCAGGTCTCTTAGTTGGCGCTTGTTACAGATTC
XII2_DW_LM_Fw	TGCCAACGTCTCATGGTCTCCTTACCGCGTCCTTGTACTGCGTC
XII2_DW_LM_Rv	TCGATCCGTCTCAGGTCTCTTTGCTGAGCGAACGTAAGAGAGG
X4_UP_AB_Fw	TGCCAACGTCTCATGGTCTCCTGCCTACCTTCATCCCTTATGTGTTC
X4_UP_AB_Rv_mut	TGCCAACGTCTCACACGTGGTGTCGAGTCAC
X4_UP_AB_Fw_mut	TGCCAACGTCTCACGTGGAAAGATCCAACTACTC
X4_UP_AB_Rv	TCGATCCGTCTCAGGTCTCTTAGTAGTGTTCCCTCAACAACC
X4_DW_LM_Fw	TGCCAACGTCTCATGGTCTCCTTACAAGAAGTAACAGGCGTGTG
X4_DW_LM_Rv	TCGATCCGTCTCAGGTCTCTTTGCTCCGCTATGTCTGAATTC
XI3_UP_AB_Fw	TGCCAACGTCTCATGGTCTCCTGCCTAGTTACTTGCTCTATGCGTTTG
XI3_UP_AB_Rv	TCGATCCGTCTCAGGTCTCCTAGTAATCAGACGCACGCTTGG
XI3_DW_LM_Fw	TGCCAACGTCTCATGGTCTCCTTACTTACGTGGATTGAGCCAGC
XI3_DW_LM_Rv	TCGATCCGTCTCAGGTCTCCTTGCTGAGAATCCGGACCAGCAG
XII5_UP_AB_Fw	TGCCAACGTCTCATGGTCTCCTGCCGCTGGTCTGCTGTAAGCAGC
XII5_UP_AB_Rv	TCGATCCGTCTCAGGTCTCCTAGTTCCTCCGGTACCGGTTCTG
XII5_DW_LM_Fw	TGCCAACGTCTCATGGTCTCCTTACACAGCAAGCAAGTTCATCATTCG
XII5_DW_LM_Rv	TCGATCCGTCTCAGGTCTCCTTGCGAACAAGGGACCTCTTTTGCC
CYP79B2_Bo_Fw	TGCCAACGTCTCATGGTCTCCAACTCTTCACCGTCGGGTAAAGATGC
CYP79B2_Bo_Rv	TCGATCCGTCTCAGGTCTCCGCTCATGAACACTTTTACCTCAAAC
Estr_SyORF_Fw	TGCCAACGTCTCATGGTCTCC
Estr_SyORF_Rv	TCGATCCGTCTCAGGTCTCC
C79_Bo_rt_Fw	ATGCTAAAGAGCCGTCCAGT
C79_Bo_rt_Rv	TTAGGGCATGTGACGGTGAT

C79_At_rt_Fw	GCCATATTTGCCACCAGGTC
C79_At_rt_Rv	ACGCAGGCAATTTCAGTGTT
C83_At_rt_Fw	GCCGTTGTTGTTTGGGCTAT
C83_At_rt_Rv	CGGCCTTCAAGTATGGCAAA
GST_At_rt_Fw	TGGCTGGTGACTTTGTTTCC
GST_At_rt_Rv	CGGTTTCTTTCCAAGCTGGT
GGP_At_rt_Fw	AGGTCATCCCGAGTACAACA
GGP_At_rt_Rv	CTTGCAGATGGTTTCCCACA
SUR_At_rt_Fw	AGCTAGAAGGGCTGTTGCTG
SUR_At_rt_Rv	AACCTGGTCTTGGCAACAAA
UGT_At_rt_Fw	GCAATTGGCCGAAGTTGCTA
UGT_At_rt_Rv	CACCAAGACACCAACAAGGC
SOT_At_rt_Fw	CCATGTGGACCTTTCTGCAC
SOT_At_rt_Rv	GGCCTTCCAGTAACCTAAGACA
ATR_At_rt_Fw	CTGCCGATGACGACCAATAC
ATR_At_rt_Rv	CGTTCTCTTCGGTGAACCAC
X3_UP_ctr_integr	TGACGAATCGTTAGGCACAG
X3_DW_ctr_integr	CCGTGCAATACCAAAATCG
XI2_UP_ctr_integr	GTTTGTAGTTGGCGGTGGAG
XI2_DW_ctr_integr	GAGACAAGATGGGGCAAG
XII2_UP_ctr_integr	CGAAGAAGGCCTCCAATTC
XII2_DW_ctr_integr	GGCCCTGATAAGGTTGTTG
X4_UP_ctr_integr	TCACAAAGGGACGAATCCTC
X4_DW_ctr_integr	GACGGTACGTTGACCAGAG
XI3_UP_ctr_integr	GTGCTTGATTTGCGTCATTC
XI3_DW_ctr_integr	CACATTGAGCGAATGAAACG
XII5_UP_ctr_integr	GCTCTTTCGTAGACGGTTTC
XII5_DW_ctr_integr	GCGATACCTTTTGTGATGGC
tADH1_ctr_integr	GTAACTCTTTCCTGTAGGTCAGG
tCYC1_ctr_integr	TTTCTGTACAGACGCGTG

Table S.3: pEM series plasmids

Plasmids obtained after each Golden gate assembly – Level 0.

The name of each pEM plasmid refers to the part present in it and to the transcriptional unit (TU) to which it will be part. P stands for promoter, A for adaptor, F for fluorescent protein, T for terminator and H for homology region. After the insert description there are two unique numbers to create a specific name for each part. L and R are references to the TU on the Left or on the Right. For example, in pEM.P01L P stands for Promoter, 01 is the number of the promoter present in the toolkit (which is p*TDH3*) and L is the reference to the TUL.

Name	Insert	Protruding ends	Primers to clone in pGA-Blue		Template
pEM.P01L	pTDH3	EF	pTDH3_EF_Fw	pTDH3_EF_Rv	CEN.PK *C* gDNA
pEM.P01R	pTDH3	FG	pTDH3_FG_Fw	pTDH3_FG_Rv	CEN.PK *C* gDNA
pEM.P02L	pENO2	EF	pENO2_EF_Fw	pENO2_EF_Rv	CEN.PK *C* gDNA
pEM.P02R	pENO2	FG	pENO2_FG_Fw	pENO2_FG_Rv	CEN.PK *C* gDNA
pEM.P03L	pPGK1	EF	pPGK1_EF_Fw	pPGK1_EF_Rv	CEN.PK *C* gDNA
pEM.P03R	pPGK1	FG	pPGK1_FG_Fw	pPGK1_FG_Rv	CEN.PK *C* gDNA
pEM.P04L	pTPI1	EF	pTPI1_EF_Fw	pTPI1_EF_Rv	CEN.PK *C* gDNA
pEM.P04R	pTPI1	FG	pTPI1_FG_Fw	pTPI1_FG_Rv	CEN.PK *C* gDNA
pEM.P05L	pCYC1	EF	pCYC1_EF_Fw	pCYC1_EF_Rv	CEN.PK *C* gDNA
pEM.P05R	pCYC1	FG	pCYC1_FG_Fw	pCYC1_FG_Rv	CEN.PK *C* gDNA
pEM.P06L	pPDA1	EF	pPDA1_EF_Fw	pPDA1_EF_Rv	CEN.PK *C* gDNA
pEM.P06R	pPDA1	FG	pPDA1_FG_Fw	pPDA1_FG_Rv	CEN.PK *C* gDNA
pEM.A01L	Adaptor	CD	ADAP_CD_Fw	ADAP_CD_Rv	pYX012 (commercial plasmid)
pEM.A01R	Adaptor	HI	ADAP_HI_Fw	ADAP_HI_Rv	pYX012 (commercial plasmid)
pEM.A02L	Adaptor	BF	ADAP_BF_Fw	ADAP_BF_Rv	pYX012 (commercial plasmid)
pEM.A02R	Adaptor	FL	ADAP_FL_Fw	ADAP_FL_Rv	pYX012 (commercial plasmid)
pEM.F01L	GFP	CD	GFP_CD_Fw	GFP_CD_Rv	Synthetic DNA (this work)
pEM.F01R	GFP	HI	GFP_HI_Fw	GFP_HI_Rv	Synthetic DNA (this work)
pEM.F02L	GFP+linker	CD	GFP+linker_CD_Fw	GFP+linker_CD_Rv	Synthetic DNA (this work)
pEM.F02R	GFP+linker	HI	GFP+linker_HI_Fw	GFP_HI_Rv	Synthetic DNA (this work)
pEM.F03L	mCherry	CD	mCh_CD_Fw	mCh_CD_Rv	pYX022-mCherry (Martani et al. 2015)
pEM.F03R	mCherry	HI	mCh_HI_Fw	mCh_HI_Rv	pYX022-mCherry (Martani et al. 2015)
pEM.T01L	tADH1	BC	tADH1_BC_Fw	tADH1_BC_Rv	pCfB3034 (Jessop-Fabre et al. 2016)
pEM.T02R	tCYC1	IL	tCYC1_IL_Fw	tCYC1_IL_Rv	pCfB3034 (Jessop-Fabre et al. 2016)
pEM.H01L	X3_UP	AB	X3_UP_AB_Fw	X3_UP_AB_Rv	pCfB3034 (Jessop-Fabre et al. 2016)
pEM.H01R	X3_DW	LM	X3_DW_LM_Fw	X3_DW_LM_Rv	pCfB3034 (Jessop-Fabre et al. 2016)
pEM.H02L	XI2_UP	AB	XI2_UP_AB_Fw	XI2_UP_AB_Rv	pCfB2903 (Jessop-Fabre et al. 2016)
pEM.H02R	XI2_DW	LM	XI2_DW_LM_Fw	XI2_DW_LM_Rv	pCfB2903 (Jessop-Fabre et al. 2016)
pEM.H03L	XII2_UP	AB	XII2_UP_AB_Fw	XII2_UP_AB_Rv	pCfB3039 (Jessop-Fabre et al. 2016)
pEM.H03R	XII2_DW	LM	XII2_DW_LM_Fw	XII2_DW_LM_Rv	pCfB3039 (Jessop-Fabre et al. 2016)
pEM.H04L	X4_UP	AB	X4_UP_AB_Fw	X4_UP_AB_Rv	pCfB3035 (Jessop-Fabre et al. 2016)
pEM.H04R	X4_DW	LM	X4_DW_LM_Fw	X4_DW_LM_Rv	pCfB3035 (Jessop-Fabre et al. 2016)
pEM.H05L	XI3_UP	AB	XI3_UP_AB_Fw	XI3_UP_AB_Rv	pCfB2904 (Jessop-Fabre et al. 2016)

pEM.H05R	XI3_DW	LM	XI3_DW_LM_Fw	XI3_DW_LM_Rv	pCfB2904 (Jessop-Fabre et al. 2016)
pEM.H06L	XII5_UP	AB	XII5_UP_AB_Fw	XII5_UP_AB_Rv	pCfB2909 (Jessop-Fabre et al. 2016)
pEM.H06R	XII5_DW	LM	XII5_DW_LM_Fw	XII5_DW_LM_Rv	pCfB2909 (Jessop-Fabre et al. 2016)
pEM.C79AtL	*AtCYP79B2*	DE	Estr_SyORF_Fw	Estr_SyORF_Rv	Synthetic DNA (this work)
pEM.C79BoL	*BoCYP79B2*	DE	CYP79B2_Bo_Fw	CYP79B2_Bo_Rv	pYX012CYP83-bTPI-CYP79 (Bartolucci et al. 2010)
pEM.C83AtR	*AtCYP83B1*	GH	Estr_SyORF_Fw	Estr_SyORF_Rv	Synthetic DNA (this work)
pEM.GSTAtL	*AtGSTF9*	DE	Estr_SyORF_Fw	Estr_SyORF_Rv	Synthetic DNA (this work)
pEM.GGPAtR	*AtGGP1*	GH	Estr_SyORF_Fw	Estr_SyORF_Rv	Synthetic DNA (this work)
pEM.SURAtL	*AtSUR1*	DE	Estr_SyORF_Fw	Estr_SyORF_Rv	Synthetic DNA (this work)
pEM.UGTAtR	*AtUGT74B1*	GH	Estr_SyORF_Fw	Estr_SyORF_Rv	Synthetic DNA (this work)
pEM.SOTAtL	*AtSOT16*	DE	Estr_SyORF_Fw	Estr_SyORF_Rv	Synthetic DNA (this work)
pEM.ATRAtR	*AtATR1*	GH	Estr_SyORF_Fw	Estr_SyORF_Rv	Synthetic DNA (this work)

Table S.4: Level 1 plasmids

Plasmids obtained after each Golden gate assembly – Level 1.

Name	pEM plasmids used as donors in the assembly										Description	Locus
**G5**	pEM.H01L	pEM.T01L	pEM.A01L	pEM.C79AtL	pEM.P04L	pEM.P03R	pEM.C83AtR	pEM.A01R	pEM.T02R	pEM.H01R	Expression of C79_*At* under TPI promoter and C83_*At* under PGK1 promoter	X-3
**G6**	pEM.H02L	pEM.T01L	pEM.A01L	pEM.SURAtL	pEM.P04L	pEM.P03R	pEM.UGTAtR	pEM.A01R	pEM.T02R	pEM.H02R	Expression of SUR_*At* under TPI promoter and UGT_*At* under PGK1 promoter	XI-2
**G7**	pEM.H03L	pEM.T01L	pEM.A01L	pEM.SOTAtL	pEM.P04L	pEM.A02R				pEM.H03R	Expression of SOT_*At* under TPI promoter	XII-2
**G8**	pEM.H04L	pEM.T01L	pEM.A01L	pEM.GSTAtL	pEM.P04L	pEM.P03R	pEM.GGPAtR	pEM.A01R	pEM.T02R	pEM.H04R	Expression of GST_*At* under TPI promoter and GGP_*At* under PGK1 promoter	X-4
**G10**	pEM.H03L	pEM.T01L	pEM.F01L	pEM.SOTAtL	pEM.P04L	pEM.A02R				pEM.H03R	Localization of SOT_*At* by GFP tagging under TPI promoter	XII-2
**G11**	pEM.H05L	pEM.A02L				pEM.P03R	pEM.ATRAtR	pEM.A01R	pEM.T02R	pEM.H05R	Expression of ATR_*At* under TPI promoter	XI-3
**G20**	pEM.H01L	pEM.T01L	pEM.A01L	pEM.C79BoL	pEM.P04L	pEM.P03R	pEM.C83AtR	pEM.A01R	pEM.T02R	pEM.H01R	Expression of C79_Bo under TPI promoter and C83_*At* under PGK1 promoter	X-3
**G22**	pEM.H01L	pEM.A02L				pEM.P03R	pEM.C83AtR	pEM.F01R	pEM.T02R	pEM.H01R	Localization of C83_*At* by GFP tagging under PGK1 promoter	X-3
**G23**	pEM.H04L	pEM.T01L	pEM.F01L	pEM.GSTAtL	pEM.P04L	pEM.A02R				pEM.H04R	Localization of GST_*At* by GFP tagging under TPI promoter	X-4
**G24**	pEM.H04L	pEM.A02L				pEM.P03R	pEM.GGPAtR	pEM.F01R	pEM.T02R	pEM.H04R	Localization of GGP_*At* by GFP tagging under PGK1 promoter	X-4
**G25**	pEM.H02L	pEM.T01L	pEM.F01L	pEM.SURAtL	pEM.P04L	pEM.A02R				pEM.H02R	Localization of SUR_*At* by GFP tagging under TPI promoter	XI-2
**G26**	pEM.H02L	pEM.A02L				pEM.P03R	pEM.UGTAtR	pEM.F01R	pEM.T02R	pEM.H02R	Localization of UGT_*At* by GFP tagging under PGK1 promoter	XI-2
**G27**	pEM.H05L	pEM.A02L				pEM.P03R	pEM.ATRAtR	pEM.F01R	pEM.T02R	pEM.H05R	Localization of ATR_*At* by GFP tagging under PGK1 promoter	XI-3
**G31**	pEM.H01L	pEM.T01L	pEM.F01L	pEM.C79BoL	pEM.P04L	pEM.A02R				pEM.H01R	Localization of C79_*Bo* by GFP tagging under TPI promoter	X-3
**G43**	pEM.H02L	pEM.T01L	pEM.F02L	pEM.SURAtL	pEM.P04L	pEM.A02R				pEM.H02R	Localization of SUR_At by GFP+linker tagging under TPI promoter	XI-2

Table S.5: Other plasmids used in this work

Plasmid name	Source
pStBlue-1	Novagen, USA
YCplac33	Gietz and Akio 1988
YCplac33_BsaIFree	This work
amilCP chromoprotein	Addgene plasmid #117847
GGE114	Addgene plasmid #120731
pGA-blue	This work
pGA-red mini	This work
pGA-red maxi	This work
pCfB2312 (TEF1p-Cas9-CYC1t_kanMX)	Jessop-Fabre et al., 2016
pCfB3041 (gRNA X-3)	Jessop-Fabre et al., 2016
pCfB3042 (gRNA X-4)	Jessop-Fabre et al., 2016
pCfB3044 (gRNA XI-2)	Jessop-Fabre et al., 2016
pCfB3045 (gRNA XI-3)	Jessop-Fabre et al., 2016
pCfB3048 (gRNA XII-2)	Jessop-Fabre et al., 2016
pCfB3051 (gRNA X-3 XI-2 XII-2)	Jessop-Fabre et al., 2016

Table S.6: Integration efficiencies for each Easy-MISE toolkit genome loci

Locus	Number of independent transformations	Number of screened colonies	Percentage of positive colonies
X-3	5	28	70%
X-4	4	16	100%
XI-2	7	79	22%
XI-3	4	21	100%
XII-2	8	36	93%
XII-5	4	45	98%
X-3/XI-2/XII-2	7	136	3%

Table S.7: Synthetic sequences

Name	Sequence
GFP_BsaFree	AGTAAAGGAGAAGAACTTTTCACTGGAGTTGTCCCAATTCTTGTTGAATTAGATGGTGATGTTAATGGGCAC<br>AAATTTTCTGTCAGTGGAGAGGGTGAAGGTGATGCAACATACGGAAAACTTACCCTTAAATTTATTTGCACT<br>ACTGGAAAACTACCTGTTCCATGGCCAACACTTGTCACTACTTTCACTTATGGTGTTCAATGCTTTTCAAGAT<br>ACCCAGATCATATGAAACGGCATGACTTTTTCAAGAGTGCCATGCCCGAAGGTTATGTACAGGAAAGAACT<br>ATATTTTTCAAAGATGACGGGAACTACAAGACACGTGCTGAAGTCAAGTTTGAAGGTGATACCCTTGTTAAT<br>AGAATCGAGTTAAAAGGTATTGATTTTAAAGAAGATGGAAACATTCTTGGACACAAATTGGAATACAACTA<br>TAACTCACACAATGTATACATCATGGCAGACAAACAAAAGAATGGAATCAAAGTTAACTTCAAAATTAGAC<br>ACAACATTGAAGATGGAAGCGTTCAACTAGCAGACCATTATCAACAAAATACTCCAATTGGCGATGGCCCT<br>GTCCTTTTACCAGACAACCATTACCTGTCCACACAATCTGCCCTTTCGAAAGATCCCAACGAAAAGAGGGAC<br>CACATGGTCCTTCTTGAGTTTGTAACAGCTGCTGGGATTACACATGGCATGGATGAACTATACAAATAA
Linker+GFP_BsaFree	GGTTCTGGTGGCTCGAGTGGTTCAAGTAAAGGAGAAGAACTTTTCACTGGAGTTGTCCCAATTCTTGTTGAA<br>TTAGATGGTGATGTTAATGGGCACAAATTTTCTGTCAGTGGAGAGGGTGAAGGTGATGCAACATACGGAAA<br>ACTTACCCTTAAATTTATTTGCACTACTGGAAAACTACCTGTTCCATGGCCAACACTTGTCACTACTTTCACT<br>TATGGTGTTCAATGCTTTTCAAGATACCCAGATCATATGAAACGGCATGACTTTTTCAAGAGTGCCATGCCC<br>GAAGGTTATGTACAGGAAAGAACTATATTTTTCAAAGATGACGGGAACTACAAGACACGTGCTGAAGTCAA<br>GTTTGAAGGTGATACCCTTGTTAATAGAATCGAGTTAAAAGGTATTGATTTTAAAGAAGATGGAAACATTCT<br>TGGACACAAATTGGAATACAACTATAACTCACACAATGTATACATCATGGCAGACAAACAAAAGAATGGAA<br>TCAAAGTTAACTTCAAAATTAGACACAACATTGAAGATGGAAGCGTTCAACTAGCAGACCATTATCAACAA<br>AATACTCCAATTGGCGATGGCCCTGTCCTTTTACCAGACAACCATTACCTGTCCACACAATCTGCCCTTTCGA<br>AAGATCCCAACGAAAAGAGGGACCACATGGTCCTTCTTGAGTTTGTAACAGCTGCTGGGATTACACATGGC<br>ATGGATGAACTATACAAATAA
AtCYP79B2	TCGATCCGTCTCAGGTCTCCGCTCATGAACACCTTCACCTCTAACTCCTCTGATTTGACTACTACTGCTACTG<br>AAACCTCTTCTTTCTCTACCTTGTACTTGTTGTCTACCTTGCAAGCTTTCGTTGCTATTACCTTGGTTATGCTG<br>CTGAAAAAGTTGATGACTGACCCAAACAAAAAGAAGCCATATTTGCCACCAGGTCCAACTGGTTGGCCAAT<br>TATTGGTATGATTCCAACCATGTTGAAGTCCAGACCAGTTTTTAGATGGTTGCACTCCATTATGAAGCAGTT<br>GAACACTGAAATTGCCTGCGTTAAGTTGGGTAACACTCATGTTATTACTGTCACCTGTCCAAAGATTGCCAG<br>AGAAATTTTGAAGCAACAAGATGCTTTGTTCGCCTCTAGACCATTGACTTATGCTCAAAAGATTCTGTCCAA<br>CGGTTACAAGACTTGTGTTATTACACCATTCGGTGACCAGTTCAAGAAGATGAGAAAAGTTGTTATGACCGA<br>ATTGGTTTGCCCAGCTAGACATAGATGGTTACACCAAAAAAGGTCCGAAGAAAACGATCATTTGACTGCTT<br>GGGTTTACAACATGGTCAAGAATTCTGGTTCTGTCGACTTCAGATTCATGACCAGACATTATTGTGGTAACG<br>CCATCAAGAAATTGATGTTCGGTACTAGAACCTTCTCTAAGAATACTGCTCCAGATGGTGGTCCAACAGTTG<br>AAGATGTTGAACATATGGAAGCTATGTTCGAAGCTTTGGGTTTTACTTTCGCTTTCTGCATCTCTGATTACTT<br>GCCAATGTTGACTGGTTTGGATTTGAATGGTCACGAGAAGATCATGAGAGAATCCTCTGCTATTATGGACAA<br>GTACCATGATCCAATTATCGACGAAAGAATCAAGATGTGGCGTGAAGGTAAGAGAACCCAAATTGAAGATT<br>TCTTGGACATCTTCATCTCCATCAAGGATGAACAAGGTAACCCATTATTGACTGCTGATGAGATTAAGCCCA<br>CCATCAAAGAATTAGTTATGGCTGCTCCTGATAACCCATCTAATGCTGTTGAATGGGCTATGGCTGAAATGG<br>TTAACAAGCCAGAAATCTTGAGAAAGGCCATGGAAGAAATCGATAGAGTTGTCGGTAAAGAAAGGTTGGTT<br>CAAGAATCCGATATTCCCAAGTTGAACTACGTTAAGGCCATTTTGAGAGAAGCCTTTAGATTGCATCCAGTT<br>GCTGCTTTTAACTTGCCTCATGTTGCTTTGTCTGATACAACTGTTGCTGGTTACCATATTCCAAAGGGTTCTC<br>AAGTTCTGTTGTCTAGATATGGTTTGGGTAGAAATCCAAAGGTTTGGGCTGATCCATTGTGTTTCAAACCAG<br>AAAGACATTTGAACGAATGCTCCGAAGTTACTTTGACCGAAAACGATTTGAGGTTCATCTCATTCTCTACTG<br>GTAAAAGAGGTTGCGCTGCTCCAGCTTTAGGTACTGCTTTAACTACTATGATGTTGGCCAGACTGTTGCAAG<br>GTTTTACATGGAAATTGCCAGAAAACGAAACCAGGGTTGAACTGATGGAATCTTCTCATGATATGTTCTTGG<br>CTAAGCCATTGGTTATGGTTGGTGATTTGAGATTACCAGAACACTTGTACCCAACCGTTAAGAGTTGGAGAC<br>CATGAGACGTTGGCA
BoCYP79B2	ATGAACACTTTTACCTCAAACTCTTCGGATCTTACTTCCACTACTAGGCAAACATGGTCGTTCAGCAACATGT<br>ATCTCCTCACGACTCTTCAAGCCTTTGTGGCTATAACCTTAGTGATGCTTCTCAAGAAAATGATCACTAATCC<br>TAATAAAAAGAAATTGTATCTCCCACCTGGACCTACCGGATGGCCCATCATCGGAATGATTCCAGCAATGCT<br>AAAGAGCCGTCCAGTTTTCCGGTGGCTCCACAGCATCATGAAGCAGCTAAACACTGAGATAGCATGCGTGA<br>GGCTAGGAAACACTAACGTGATCACCGTCACATGCCCTAAGATAGCACGTGAGATACTCAAGCAACAAGAC<br>GCTCTCTTCGCCTCAAGACCTATGACTTACGCGCAAAACGTCCTCTCTAACGGATACAAAACCTGCGTGATC<br>ACTCCGTTCGGGGAACAATTCAAGAAAATGAGGAAAGTCGTGATGACGGAACTCGTTTGTCCGGCGAGACA<br>CAGGTGGCTTCATCAGAAGAGAGCGGAAGAAAACGACCATTTAACCGCATGGGTATACAACATGGTTAAGA<br>ACTCGGGCTCAGTCGATTTCCGGTTTGTGACAAGGCATTACTGCGGAAATGCTATCAAGAAACTTATGTTCG<br>GGACAAGAACGTTCTCTGAAAACACTGCAGCGGACGGTGGGCCAACCGCCGAGGATATCGATCATATGGAA<br>GCTATGTTTGAAGCATTAGGCTTTACGTTTGCTTTTTGTATATCTGATTATCTACCTATGCTCACGGGACTTG<br>ATCTTAACGGTCACGAGAAGATCATGAGAGATTCAAGTGCTATTATGGACAAGTATCACGATCCTATCATTG<br>ATGGAAGGATCAAAATGTGGAAAGAAGGAAAGAGAACTCAAATCGAGGATTTTCTAGATATTTTCATTTCG<br>ATCAAAGATGAAGAAGGCAACCCATTGCTTACCGCTGATGAAATCAAACCCACTATTAAGGAGCTTGTAAT<br>GGCGGCGCCAGACAATCCATCAAACGCCGTAGAATGGGCCATGGCGGAGATGGTAAACAAACCTGAGATA<br>CTCCGTAAGGCAATGGAAGAAATAGACAGAGTGGTCGGAAAAGAAAGACTTGTCCAAGAATCCGACATCC<br>CAAAACTAAACTACGTCAAAGCTATTCTCCGTGAAGCTTTCCGTCTCCATCCTGTCGCCGCCTTTAACCTCCC<br>ACACGTGGCACTTTCCGACACAACCGTCGCCGGATATCACATCCCTAAAGGAAGTCAAGTACTTCTCAGTCG<br>ATATGGGCTGGGCCGTAACCCAAAAGTTTGGGCCGACCCACTTAGCTTTAAACCGGAGAGACATCTCAATG<br>AATGCTCAGAAGTTACTTTGACGGAGAACGATCTCCGTTTTATCTCGTTTAGCACCGGGAAAAGAGGTTGTG<br>CTGCTCCGGCTTTAGGTACAGCGTTGACTACGATGATGCTCGCAAGACTTCTTCAAGGTTTCACTTGGAAGC<br>TACCGGAGAATGAAACACGTGTTGAGCTGATGGAGTCTAGTCATGATATGTTTTTGGCTAAACCGTTGGTTA<br>TGGTAGGTGAGTTGAGATTGCCGGAGCATCTTTACCCGACGGTGAAG
AtCYP83B1	TGCCAACGTCTCATGGTCTCCATTCATGGACCTGTTGTTGATTATTGCTGGTTTGGTTGCTGCTGCTGCATTTT<br>TCTTTTTGCGTTCTACTACCAAGAAGTCCTTGAGATTGCCACCAGGTCCAAAAGGTTTGCCAATTATTGGTAA<br>CTTGCACCAGATGGAAAAGTTCAACCCACAACATTTCTTGTTCAGGCTGTCTAAGTTGTACGGTCCAATTTTC<br>ACTATGAAGATCGGTGGTAGAAGATTGGCCGTTATTTCTTCTGCTGAATTGGCTAAAGAGCTGTTGAAAACT<br>CAAGACTTGAACTTCACTGCTAGACCTTTGTTGAAAGGTCAACAAACCATGTCTTACCAGGGTAGAGAATTA<br>GGTTTTGGTCAGTACACTGCTTACTACAGGGAAATGAGAAAGATGTGCATGGTCAATTTGTTCTCCCCAAAT<br>AGAGTTGCTTCTTTCAGACCAGTTAGAGAAGAGGAATGTCAAAGAATGATGGACAAGATCTACAAGGCTGC<br>TGATCAATCTGGTACTGTTGATTTGTCTGAGCTGTTGTTGTCTTTCACTAACTGTGTTGTATGCAGACAAGCT

	TTCGGTAAGAGATACAATGAATACGGTACTGAGATGAAGAGGTTCATCGATATCTTGTACGAAACCCAAGC<br>TTTGTTGGGTACTTTGTTCTTCTCTGATTTGTTCCCATACTTCGGTTTCTTGGATAACTTGACTGGTTTGTCTG<br>CTAGATTGAAGAAGGCTTTCAAAGAATTGGACACCTACTTGCAAGAACTGTTGGACGAAACTTTGGATCCTA<br>ATAGACCAAAGCAAGAGACTGAATCCTTCATCGATTTGTTGATGCAGATCTATAAGGACCAGCCATTCTCTA<br>TTAAGTTCACTCACGAAAACGTTAAGGCCATGATCTTGGATATAGTTGTTCCAGGTACTGATACTGCAGCTG<br>CCGTTGTTGTTTGGGCTATGACTTATTTGATTAAGTACCCAGAGGCTATGAAGAAAGCCCAAGATGAAGTTA<br>GATCTGTTATCGGTGATAAGGGTTACGTGTCCGAAGAAGATATTCCAAATTTGCCATACTTGAAGGCCGTCA<br>TCAAAGAAAGTTTGAGATTGGAACCAGTCATCCCAATCTTGTTGCATAGAGAAACTATTGCCGATGCTAAGA<br>TTGGTGGTTATGATATTCCAGCCAAGACCATCATTCAAGTTAATGCTTGGGCTGTTTCTAGAGATACTGCTGC<br>TTGGGGTGATAATCCAAACGAATTCATTCCAGAACGTTTCATGAACGAACACAAGGGTGTTGATTTTAAGGG<br>TCAAGATTTTGAGTTGTTGCCATTCGGTTCAGGTAGAAGAATGTGTCCAGCTATGCATTTGGGTATTGCCAT<br>GGTTGAAATTCCATTCGCCAATCTGTTGTACAAGTTCGATTGGTCTTTGCCAAAAGGTATTAAGCCAGAGGA<br>TATCAAGATGGATGTTATGACAGGTTTGGCCATGCATAAGAAAGAACATTTGGTTTTGGCCCCAACTAAGCA<br>CATTACCGGGAGACCTGAGACGGATCGA
AtGSTF9	TCGATCCGTCTCAGGTCTCCGCTCATGGTCTTGAAAGTTTACGGTCCACATTTTGCTTCTCCAAAGAGAGCTT<br>TGGTTACCTTGATTGAAAAGGGTGTTGCTTTCGAAACCATTCCAGTTGATTTGATGAAGGGTGAACATAAGC<br>AACCAGCTTACTTGGCTTTACAACCATTTGGTACTGTTCCAGCTGTTGTTGATGGTGATTACAAGATCTTTGA<br>ATCCAGAGCCGTTATGAGATACGTTGCTGAAAAGTATAGATCCCAAGGTCCAGATTTGTTGGGTAAGACTGT<br>TGAAGATAGAGGTCAAGTTGAACAATGGTTGGATGTTGAAGCTACTACTTACCATCCACCACTGTTGAATTT<br>GACCTTGCATATTATGTTCGCCTCTGTTATGGGTTTTCCATCTGACGAAAAGCTGATCAAAGAATCCGAAGA<br>AAAATTGGCCGGTGTTTTGGATGTTTACGAAGCTCATTTGTCCAAGTCTAAATACTTGGCTGGTGACTTTGTT<br>TCCTTGGCTGATTTGGCTCATTTGCCATTCACTGATTATTTGGTTGGTCCAATTGGTAAGGCCTACATGATCA<br>AGGATAGAAAACATGTTAGTGCCTGGTGGGATGATATTTCTTCTAGACCAGCTTGGAAAGAAACCGTTGCTA<br>AATATTCTTTCCCAGCTAGTTGGAGACCATGAGACGTTGGCA
AtGGP1	TGCCAACGTCTCATGGTCTCCATTCATGGTCGAACAAAAGAGATACGCTTTGTTCTTGGCTACCTTGGATTCT<br>GAATTCGTCAAAAAAACTTACGGTGGTTACCACAACGTTTTCGTTACTACTTTTGGTGATGAAGGTGAACAC<br>TGGGATTCTTTCAGAGTTGTTTCTGGTGAATTTCCAGACGAAAAGGACTTGGAAAAGTACGATGGTTTCGTT<br>ATCTCTGGTTCTTCTCATGATGCTTTCGAAAACGATGATTGGATCTTGAAGTTGTGCGACATCGTTAAGAAA<br>ATCGACGAGATGAAGAAGAAGATCTTGGGTATTTGTTTCGGCCATCAAATTATCGCTAGAGTTAGAGGTGGT<br>ACTGTTGGTAGAGCTAAAAAAGGTCCAGAATTGAAGTTGGGTGATATTACCATTGTCAAGGATGCTATTACT<br>CCAGGTTCTTACTTCGGTAACGAAATCCCAGATTCCATTGCCATTATTAAGTGCCACCAAGATGAAGTTTTG<br>GTCTTGCCAGAAACTGCTAAAGTTTTGGCTTACTCCAAGAACTACGAAGTCGAAATGTACTCCATCGAAGAT<br>CATTTGTTCTGCATTCAAGGTCATCCCGAGTACAACAAAGAAATCTTGTTCGAAATCGTCGATAGAGTTTTG<br>GCATTGGGTTACGTTAAGCAAGAATTTGCTGATGCTGCTAAAGCTACCATGGAAAACAGAGGTGCTGATAG<br>AAAATTGTGGGAAACCATCTGCAAGAACTTTTTGAAAGGTAGAGTCCCTACCAACACCGGGAGACCTGAGA<br>CGGATCGA
AtSUR1	TCGATCCGTCTCAGGTCTCCGCTCATGTCCGAAGAACAACCACATGCTAATTTGGCTGTTCCAGCTTTCAAA<br>ACCGAAAAAGAACCTATCACTCAAACCAGAAACGGCCAATCTTCTGTTTGGAGATTTGGTGGTTCTGATAAG<br>GCTGCTAAAGCTTCTACTGTTACTTTGAGAGGTGTCATCTACATGTTGTTCGATAACTGTGGTAAGGATGTCA<br>ACAAGACCATTTTGCCATTAGGTCATGGTGATCCATCTGTTTACCCATGTTTCAGAACTTGCATTGAAGCTGA<br>AGATGCCGTTGTTGATGTTTTGAGATCTGGTAAGGGTAATTCTTATGGTCCAGGTGCTGGTATTTTGCCAGCT<br>AGAAGGGCTGTTGCTGATTACATGAATAGAGATTTGCCACATAAGTTGACCCCAGAGGATATTTTCTTGACT<br>GCTGGTTGTAATCAGGGTATCGAAATCGTTTTTGAATCCTTGGCTAGACCAAACGCCAATATTTTGTTGCCA<br>AGACCAGGTTTTCCACATTATGATGCTAGAGCTGCTTACTCTGGTTTGGAAGTTAGAAAGTTTGACCTGTTG<br>CCAGAAAAAGAATGGGAAATTGATTTGGAAGGTATCGAAGCTATTGCTGACGAAAACACTGTTGCTATGGT<br>TGTTATTAACCCAAACAACCCATGTGGTAACGTTTACTCTCATGATCACTTGAAGAAGGTTGCTGAAACTGC<br>TAGAAAGTTGGGTATCATGGTTATCTCCGATGAAGTTTACGATAGAACCATCTTTGGTGACAACCCATTTGT<br>CTCTATGGGTAAATTTGCTTCCATCGTTCCAGTTTTGACTTTGGCCGGTATTTCTAAAGGTTGGGTTGTTCCA<br>GGTTGGAAGATTGGTTGGATTGCTTTGAATGATCCAGAAGGTGTTTTCGAAACTACCAAGGTATTGCAATCC<br>ATCAAGCAAAACTTGGATGTTACTCCAGATCCAGCCACTATTATTCAAGCTGCTTTACCAGCTATTTTGGAA<br>AAGGCTGATAAGAACTTCTTCGCCAAGAAGAACAAGATCCTGAAGCACAATGTTGATTTGGTTTGCGATAG<br>ATTGAAGGATATCCCATGTGTTGTTTGTCCAAAGAAGCCAGAATCTTGTACTTACTTGTTGACCAAGTTGGA<br>GTTGTCTTTGATGGATAACATCAAGGACGATATCGATTTCTGCGTTAAGTTGGCTAGAGAAGAGAACTTGGT<br>TTTTTTGCCAGGTGATGCTTTGGGTTTGAAGAACTGGATGAGAATTACCATTGGTGTTGAAGCCCACATGTT<br>AGAAGATGCATTGGAAAGATTGAAAGGTTTCTGTACCAGACATGCCAAAAAGACTGAAACTGAAACCGAAT<br>CATTGCAGGCTTTGAAGTTGTCTGATAACAACTTGGAGATGAGTTGGAGACCATGAGACGTTGGCA
AtUGT74B1	TGCCAACGTCTCATGGTCTCCATTCATGGCTGAAACTACTCCAAAGGTTAAGGGTCATGTTGTCATTTTGCCA<br>TATCCAGTTCAAGGTCATTTGAACCCAATGGTTCAATTCGCTAAGAGGTTGGTTTCTAAGAACGTTAAGGTT<br>ACTATTGCCACTACTACTTACACCGCTTCTTCTATTACTACCCCATCTTTGTCTGTTGAACCTATCTCTGATGG<br>TTTCGATTTCATTCCAATTGGTATCCCAGGTTTCTCCGTTGATACTTACTCTGAATCTTTCAAGTTGAACGGCT<br>CTGAAACTTTGACCTTGTTGATCGAAAAGTTCAAGTCTACCGATTCTCCAATTGACTGCTTGATCTACGATTC<br>TTTTTTGCCATGGGGTTTAGAAGTTGCCAGATCTATGGAATTGTCTGCTGCTTCTTTTTTCACCAACAACTTG<br>ACCGTTTGCTCCGTTTTGAGAAAGTTTTCTAATGGTGATTTCCCATTGCCAGCTGATCCAAATTCTGCTCCTT<br>TTAGAATTAGAGGCTTGCCCTCTTTGTCTTACGATGAATTGCCATCTTTTGTTGGTAGACATTGGTTGACTCA<br>TCCAGAACATGGTAGAGTCTTGTTGAATCAATTTCCAAACCACGAAAACGCCGATTGGTTGTTTGTTAATGG<br>TTTTGAAGGTCTGGAAGAAACCCAAGATTGTGAAAATGGTGAATCCGATGCTATGAAGGCTACTTTGATTGG<br>TCCAATGATTCCATCTGCTTACTTGGATGATAGAATGGAAGATGATAAGGATTACGGTGCCTCTTTGTTGAA<br>GCCAATTTCTAAAGAATGCATGGAATGGTTGGAAACTAAGCAAGCTCAATCTGTTGCTTTCGTTTCCTTTGG<br>TTCTTTCGGTATCTTGTTCGAAAAGCAATTGGCCGAAGTTGCTATTGCATTGCAAGAATCTGATCTGAACTTC<br>CTGTGGGTTATCAAAGAAGCTCATATTGCCAAATTGCCAGAAGGTTTCGTTGAATCTACAAAGGATAGAGCC<br>TTGTTGGTGTCTTGGTGTAATCAACTAGAAGTTTTGGCCCATGAATCTATCGGTTGTTTCTTGACTCATTGTG<br>GTTGGAACTCTACTTTGGAAGGTTTGTCTTTGGGTGTTCCAATGGTTGGTGTTCCACAATGGTCTGATCAAAT<br>GAATGATGCCAAGTTCGTTGAAGAAGTTTGGAAAGTTGGTTACAGAGCTAAAGAAGAAGCTGGCGAAGTTA<br>TCGTTAAGTCCGAAGAATTGGTTAGATGCTTGAAGGGTGTTATGGAAGGTGAATCCTCTGTTAAGATCAGGG<br>AATCTTCTAAGAAGTGGAAAGATTTGGCTGTTAAGGCTATGTCTGAAGGTGGTTCTTCTGACAGATCTATCA<br>ACGAATTCATCGAATCCTTGGGCAAGACCGGGAGACCTGAGACGGATCGA
AtSOT16	TCGATCCGTCTCAGGTCTCCGCTCATGGAATCCAAGACTACTCAAAACGGTTCCGAAGTTGTTGAATTGACC<br>GAATTCGAAAAGACCCAAAAGAAGTACCAAGATTTCATTGCTACCCTGCCAAAATCTAAAGGTTGGAGGCC<br>AGACGAAATTTTGACTCAATATGGTGGTCATTGGTGGCAAGAATGTTTGTTGGAAGGTTTGTTTCATGCCAA<br>GGATCATTTTGAAGCTAGACCAACTGATTTCTTGGTTTGCTCTTATCCAAAGACTGGTACTACTTGGTTGAAG

	GCTTTGACTTACGCTATCGTTAACAGATCCAGATATGATGATGCTGCTAACCCTTTGTTGAAGAGAAATCCA<br>CATGAATTCGTTCCATACGTCGAAATTGATTTCGCTTTCTACCCAACTGTTGATGTGTTGCAAGATAGAAAG<br>AATCCCTTGTTCTCTACCCATATTCCAAATGGTTTGTTGCCAGATTCCATCGTTAATTCCGGTTGTAAGATGG<br>TCTACATTTGGAGAGATCCAAAGGATACCTTCATTTCCATGTGGACCTTTCTGCACAAAGAAAAGTCTCAAG<br>AAGGTCAATTGGCCTCCTTGGAAGATTCTTTTGATATGTTCTGTAAGGGCTTGTCTGTTTACGGTCCATATTT<br>GGATCATGTCTTAGGTTACTGGAAGGCCTATCAAGAAAACCCAGATAGAATCTTGTTCCTGAGATACGAAA<br>CTATGAGAGCTAATCCATTGCCATTCGTTAAGAGATTGGCTGAATTCATGGGTTACGGTTTTACTGATGAAG<br>AAGAAGAAAACGGCGTTGCTGAAAAGGTTGTTAAGTTGTGTTCTTTCGAAACCCTGAAGAACTTGGAAGCT<br>AACAAAGGTGACAAAGAAAGAGAAGATAGACCAGCTGTTTACGCTAATTCTGCTTACTTCAGAAAAGGTAA<br>GGTTGGTGATTGGGCTAATTACTTGACTCCAGAAATGGCTGCTAGAATCGATGGTTTAGTTGAGGAAAAGTT<br>CAAGGATACTGGTCTGTTGCAGCATGATAACAGTTGGAGACCATGAGACGTTGGCA
AtATR1	TGCCAACGTCTCATGGTCTCCATTCATGACTTCTGCCTTGTATGCCTCTGATTTGTTCAAGCAATTGAAGTCC<br>ATTATGGGCACCGATTCTTTGTCTGATGATGTTGTTTTGGTTATCGCTACTACCTCTTTGGCTTTGGTTGCTGG<br>TTTTGTTGTTCTGTTGTGGAAAAAGACTACCGCTGATAGATCTGGTGAATTGAAACCATTGATGATCCCCAA<br>ATCTTTGATGGCCAAAGATGAAGATGATGACTTGGACTTAGGTTCTGGTAAGACTAGAGTTTCCATTTTCTT<br>CGGTACTCAAACTGGTACTGCTGAAGGTTTTGCTAAAGCTTTGTCCGAAGAAATCAAGGCCAGATACGAAA<br>AAGCTGCCGTTAAGGTTATTGATTTGGATGATTATGCTGCCGATGACGACCAATACGAAGAAAAGTTGAAG<br>AAAGAAACCTTGGCCTTCTTCTGTGTTGCTACTTATGGTGATGGTGAACCTACTGATAATGCTGCTAGATTTT<br>ACAAGTGGTTCACCGAAGAGAACGAAAGAGATATCAAGTTGCAACAATTGGCCTACGGTGTTTTTGCTTTG<br>GGTAATAGACAATACGAGCACTTCAACAAGATCGGTATCGTTTTGGATGAAGAGTTGTGTAAAAAGGGTGC<br>CAAGAGATTGATTGAAGTTGGTTTGGGTGATGATGACCAGTCTATCGAAGATGATTTTAACGCCTGGAAAG<br>AATCCTTGTGGTCTGAATTGGATAAGTTGTTGAAGGACGAAGATGACAAATCTGTTGCTACACCATACACTG<br>CTGTTATTCCAGAGTATAGAGTTGTTACTCACGATCCAAGATTCACGACTCAAAAGTCTATGGAATCTAACG<br>TTGCTAACGGTAACACCACCATCGATATTCATCATCCATGTAGAGTTGATGTCGCCGTCCAAAAAGAATTGC<br>ATACTCATGAATCCGACAGATCCTGCATTCATTTGGAATTCGATATTTCCAGAACCGGTATTACTTACGAAA<br>CCGGTGATCATGTTGGTGTTTACGCTGAAAATCACGTTGAAATCGTTGAAGAAGCCGGTAAGTTGTTAGGTC<br>ATTCATTGGATTTGGTGTTCTCCATTCATGCCGACAAAGAAGATGGTTCTCCTTTGGAATCTGCTGTTCCACC<br>ACCATTTCCAGGTCCATGTACTTTAGGTACTGGTTTGGCTAGATATGCTGACTTGTTGAATCCACCAAGAAA<br>GTCTGCTTTAGTTGCTTTGGCTGCTTATGCTACTGAACCATCTGAAGCCGAAAAATTGAAACATTTGACTTCC<br>CCAGATGGTAAGGACGAATATTCTCAATGGATAGTTGCCTCTCAGAGGTCTTTGTTGGAAGTTATGGCTGCT<br>TTTCCATCTGCTAAACCACCATTGGGTGTTTTTTTTGCTGCTATTGCTCCAAGATTGCAACCTAGGTATTACT<br>CCATTTCTTCATCACCAAGATTGGCCCCATCTAGAGTTCATGTTACATCTGCTTTGGTTTATGGTCCAACTCC<br>AACTGGTAGAATTCATAAGGGTGTTTGTTCTACCTGGATGAAGAACGCTGTTCCAGCTGAAAAATCTCATGA<br>ATGTTCTGGTGCCCCAATTTTCATTAGAGCTTCTAATTTCAAGCTGCCAAGCAATCCATCTACTCCAATAGTT<br>ATGGTTGGTCCAGGTACAGGTTTAGCTCCTTTTAGAGGTTTCCTACAAGAAAGGATGGCCTTGAAAGAGGAT<br>GGCGAAGAATTGGGTTCTTCCTTGTTGTTTTTTGGTTGCAGAAACAGACAGATGGATTTCATCTATGAGGAC<br>GAGTTGAACAACTTCGTTGATCAAGGTGTTATCTCCGAATTGATTATGGCCTTTTCTAGAGAAGGTGCCCAG<br>AAAGAATATGTCCAACATAAGATGATGGAAAAAGCCGCTCAAGTTTGGGACCTAATCAAAGAAGAAGGAT<br>ACTTGTACGTTTGCGGTGATGCTAAAGGTATGGCTAGAGATGTTCATAGAACATTGCATACCATCGTCCAAG<br>AACAAGAAGGTGTTTCATCTTCTGAAGCTGAAGCTATCGTTAAGAAGTTGCAAACTGAAGGTAGATACTTG<br>AGAGATGTCTGGACCGGGAGACCTGAGACGGATCGA
